# Economic effectiveness of pharmacogenomics-guided prescribing for psychiatric disorders: a systematic review and meta-analysis

**DOI:** 10.1038/s41397-026-00408-2

**Published:** 2026-04-04

**Authors:** Ellen R. Mason, Mohamed Y. Ali, David S. Gibson, Elaine K. Murray, Richéal M. Burns, Catriona Kelly

**Affiliations:** 1 Personalised Medicine Centre, School of Medicine, Ulster University, C-TRIC Builiding, Altanagelvin Hospital Campus, Glenshane Road, Derry∼Londonderry, BT47 6SB UK; 2 Department of Health and Nutritional Science, Atlantic Technological University, Faculty of Science, Sligo, F91 YW50 Ireland; 3 Health and Biomedical Research Centre (HEAL), Atlantic Technological University, Faculty of Science, Sligo, F91 YW50 Ireland

**Keywords:** Pharmacogenomics, Personalized medicine

## Abstract

Pharmacogenomics (PGx)-guided prescribing is a promising approach to reduce variability in drug response, although its cost-effectiveness remains uncertain. We performed a systematic review and meta-analysis evaluating the cost-effectiveness of PGx-guided prescribing compared to standard care in psychiatry. In January 2026, we searched MEDLINE, Embase and PsycINFO for studies published between 2014 and 2025. We included any peer-reviewed study that included adults with a diagnosed psychiatric disorder, comparing PGx-guided prescribing to standard care, and reported both quality-of-life and economic outcomes. Given the lack of consensus on synthesising economic evidence, both a narrative synthesis and meta-analysis were conducted. Pooled incremental net benefit (INB) was used as the effect measure for the meta-analysis and heterogeneity measures including the I^2^ test were used to assess heterogeneity and determine which model to use for the meta-analysis. From an initial 1 271 records, 17 studies were included. The narrative synthesis found that 88% of studies favoured PGx-guided prescribing. Meta-analyses produced positive, though non-significant, pooled Incremental Net Benefits (INBs) for the total study groups (£1 623.14, 95% CI: -£116.50 to £3 362.79, *p* = 0.07, I^2^ = 100%), and for a statistically homogeneous subgroup (£41.54, 95% CI: -£18.27 to £101.35, *p* = 0.17, I^2^ = 0%). Our review indicates that PGx-guided prescribing can be cost-effective in psychiatry but highlights the need for increased consensus in economic modelling methods.

## Introduction

Psychiatric disorders are a collection of complex conditions that affect an individual’s mood, thoughts and behaviour. In 2021, these conditions affected over 1 billion people worldwide [1, 2]. Treatment for these disorders typically involves a combination of psychotherapy and pharmacotherapy, although long waiting times for psychological interventions often results in pharmacological treatments becoming the first-line therapy [3]. Despite pharmacotherapy being a first-line approach to treatment, ∼20–60% of patients do not respond adequately to these medications and so are considered treatment-resistant [4]. This resistance to treatment reflects the inter-individual heterogeneity in clinical, demographic, lifestyle and genetic factors and highlights the limitations of non-personalised prescribing. Among these factors, genetic variation, which can particularly influence pharmacokinetics, is estimated to account for around 15–30% of this variation alone [5]. This has led to growing interest in using personalised medicine approaches to psychiatric prescribing to improve treatment outcomes.

Pharmacogenomics (PGx) integrates the subjects of pharmacology and genomics to understand how genetic variations can influence drug metabolism, efficacy, and the occurrence of adverse drug reactions (ADRs) [6]. Genetic variations can affect drug response when they occur in genes that encode proteins involved in drug metabolism as they can alter the rate at which an individual metabolises drugs. A family of proteins that play a key role in drug metabolism are the cytochrome p450 (CYP450) enzymes [7]. Understanding these genetic variations has led to the classification of individuals into various metaboliser phenotypes including poor, intermediate, normal and ultra-rapid metabolisers. A recent randomised controlled trial (RCT) found that 93.5% of their study population had at least one genetic variant that affected their response to one or more of the 39 evaluated medications [8]. Utilising these phenotypes to guide drug allocation and dosing could enable stratified prescribing for patient subgroups who would be most likely to benefit, or for those at increased risk of ADRs. Prescribing recommendations according to these phenotypes have already been developed for various antidepressants and antipsychotics by PGx organisations such as the Dutch Pharmacogenetics Working Group and the Clinical Pharmacogenetics Implementation Consortium [9–13].

Despite the development of these prescribing recommendations, numerous barriers are hindering the implementation of PGx-guided prescribing. These include: the perceived complexity of PGx to healthcare providers; concerns about disruption to the current workflow for prescribers; limitations in the usability of clinical decision support (CDS) systems; insufficient IT infrastructure to facilitate result delivery; and the cost-effectiveness of PGx-informed prescribing compared to current prescribing practices [14]. Cost-effectiveness is a key factor which will inform the decision of whether PGx-informed prescribing can be implemented into routine practice. For an intervention to be adopted it must highlight the potential to be both clinically efficacious and cost-effective. A 2021 systematic review by Karamperis and colleagues was the first comprehensive assessment of studies economically evaluating PGx within psychiatry. Their results showed 16 out of 18 (89%) studies favored PGx-guided prescribing [15]. This review builds on the narrative synthesis by Karamperis and colleagues by conducting meta-analyses of included studies, offering the first quantitative synthesis on the cost-effectiveness of PGx-guided prescribing in psychiatry.

Given the rapidly evolving awareness and knowledge of PGx, alongside advancements in clinical delivery models and CDS systems, there is a need for ongoing review to evaluate the cost-effectiveness of implementing PGx-led prescribing. Therefore, the aim of this systematic review was to provide an up-to-date assessment of the literature and to conduct a quantitative analysis to answer whether PGx-guided prescribing of medications for psychiatric disorders is more cost-effective than standard care.

## Methods

### Search strategy and study selection

We conducted a systematic review and meta-analysis of eligible studies identified in a literature search of the databases MEDLINE, Embase, and PsycINFO between 2014 and 2025. The search was initially conducted in December 2024 and updated in January 2026 to include studies published in 2025 (search strategy in Supplementary Tables 1 and 2). Studies were considered eligible using our search criteria if they had: (1) an adult population clinically diagnosed with any psychiatric disorder covered by the international classification of diseases, tenth revision (ICD-10), chapter V; (2) were evaluating PGx-guided prescribing compared to standard care; and (3) included both health-related quality of life and cost outcomes. Covidence was used for the screening process and any duplicates were removed using the built-in deduplication algorithm [16]. Title and abstract screening were performed by one reviewer (ERM) and full-text screening by two independent reviewers (ERM and MYA) and any discrepancies were resolved through discussion. We excluded any studies that did not meet our inclusion criteria (Supplementary Methods), were not peer-reviewed, or were not in English. We also excluded any studies using a pre-post PGx-testing or incongruent versus congruent prescribing study design. PRISMA guidelines were followed for the review (Supplementary Tables 3 and 4) and the review protocol was registered with PROSPERO (CRD42024622045, Supplementary Methods).

### Data extraction

Data was manually extracted from full-text versions of all included studies independently by two reviewers (ERM and MYA). The data extraction form used was adapted from the step-by-step process to harmonise data from cost-utility analyses, proposed by Bagepally and colleagues [17]. This data extraction form covers all relevant data for comparing economic evaluations including general study characteristics, article information, participant characteristics, study methods and findings. The primary measure for cost-effectiveness that was extracted was the Incremental Cost-Effectiveness Ratio (ICER).

The ICER is used to compare a new intervention with the current standard of care by showing the extra cost required to achieve an additional health benefit. It is calculated by dividing the difference in the costs by the difference in their health benefits. Health benefits are commonly measured using quality-adjusted life years (QALYs). One QALY represents one year in perfect health, while negative values indicate reduced health-related quality of life [18]. For any studies that did not report their ICER values (*n* = 7), this value was estimated using the data provided. Cost and QALY data used for the meta-analysis were extracted using WebPlotDigitizer from any model-based studies which reported Cost-Effectiveness (CE) planes produced from a Probabilistic Sensitivity Analysis (PSA) [19].

### Quality appraisal

The Quality of Health Economic Studies (QHES) checklist was used for quality assessment [20]. This was carried out independently by two reviewers (ERM and MYA) and consensus was reached through discussion. Some of the QHES questions are particularly relevant for model-based studies. For any studies that were not model-based but had the relevant outcomes, questions were adapted accordingly (Supplementary Table 5). Certainty of evidence was assessed using the five GRADE domains: risk of bias; indirectness; inconsistency; imprecision; and publication bias [21]. Each domain was explored narratively, and results from the funnel plots and Egger’s regression-based tests were used to assess publication bias.

### Data synthesis and meta-analysis

Given the ambiguity in interpreting negative ICER values, where a negative ICER can indicate that the new intervention is less costly or that it is less effective, we did not use it as the effect measure for our meta-analysis. Instead, Incremental Net Benefit (INB) was calculated using data extracted from the CE planes. The INB also compares costs and health benefits between interventions, but unlike the ICER, it expresses the benefits in monetary terms and therefore a negative INB indicates that the new intervention is not cost-effective [17]. For our meta-analysis we only included model-based studies to ensure comparability between studies. To ensure comparability of cost estimates across studies, costs were converted to a common currency and year using the ‘CCEMG–EPPI Centre Cost Converter’ (v.1.7 last update: January 2024) [22]. This tool accounts for factors such as inflation and currency differences, placing all costs on a comparable scale. All monetary units were adjusted to 2024 Great British Pounds (GBP).

The INB and its variance were then calculated using the following equations:

$${INB}=K\times \Delta E-\Delta C$$

$${Var}\left({INB}\right)={K}^{2}{\sigma }_{\Delta E\,}^{2}+{\sigma }_{\Delta C}^{2}-2K{\sigma }_{\Delta E\Delta C}$$

Where *K* = Willingness-to-pay (WTP) threshold, Δ*E* = Incremental effect, Δ*C* = Incremental cost,$\,{\sigma }_{\Delta E}^{2}$ = Variance of incremental effect, ${\sigma }_{\Delta C}^{2}$ = Variance of incremental cost and ${\sigma }_{\Delta E\Delta5 C}$ = Covariance between incremental costs and incremental effect.

If heterogeneity was present (I^2^ ≥ 25%), a random-effects model was used. Restricted Maximum Likelihood was used to estimate variance as it is preferred when there are small study numbers and there is high variability [23]. Otherwise, a fixed-effect model was applied following the method by Bagepally and colleagues [17]. Results from the meta-analysis were visualised using forest plots. Heterogeneity was evaluated using various statistical tests, Galbraith plots and exploratory subgroup analyses. Galbraith plots were used to visualise heterogeneity and to identify potential outliers. Our subgroup analyses were only exploratory due to the small number of studies in our meta-analysis which would lack enough power for a formal subgroup analysis [24, 25]. A leave-one-out analysis was then used to assess the influence of potential outliers on the overall effect size. Publication bias was evaluated using funnel plots and the Egger’s regression-based test. All statistical analyses were performed using Microsoft Excel (Version 2505 Build 16.0.18827.20102) and IBM SPSS Statistics (Version 29.0.2.0 (20)).

## Results

**Fig. 1:**
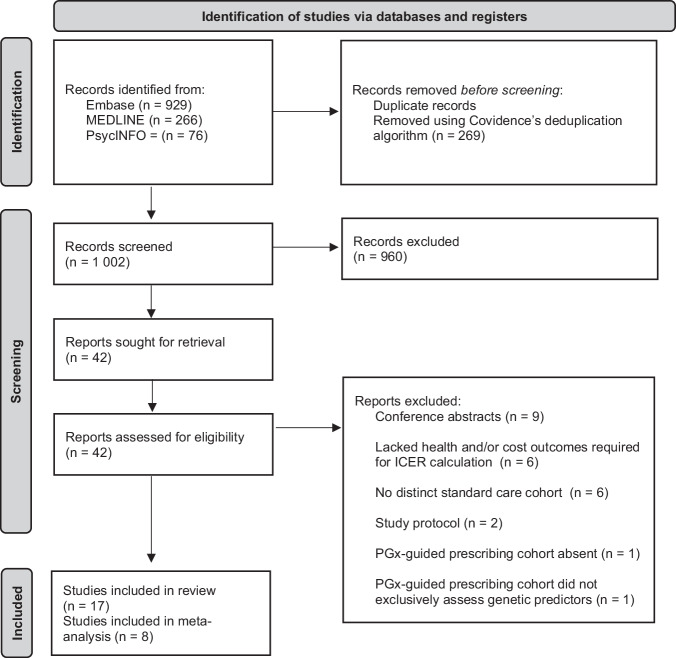
Study selection. *ICER* Incremental Cost-Effectiveness Ratio, *PGx* Pharmacogenomics Of the 1 271 unique identified studies, 42 full-text articles were assessed for eligibility, 17 were included in the final analysis and 11 study groups from 8 studies in the meta-analysis.

In total, the literature search identified 1 271 articles. Seventeen studies met the inclusion criteria for our systematic review and 11 study groups from eight studies were included in the meta-analysis (Fig. 1) [26–42]. Excluded studies and reasons for exclusion are provided in Supplementary Table 6.

The summary of study characteristics and strategies reveals a predominance of recent research into the cost-effectiveness of PGx-informed prescribing with 59% (*n* = 10) published between 2019–2025. Research was primarily from a European perspective (*n* = 9, 53%) followed by a United States (US) perspective (*n* = 4, 24%). Depression and schizophrenia were the most common disorders studied, accounting for 70% (*n* = 12) and 24% (*n* = 4) of the studies respectively. Studies with cohorts defined as Major Depressive Disorder (MDD) or moderate-to-severe depression were collectively considered depression. Most studies explored a multi-gene approach to PGx-led prescribing (*n* = 10, 59%), six (35%) evaluated single-gene testing, and one study looked at both single- and multi-gene testing, as they simulated cohorts from the UK and Japan, reflecting genetic differences between populations [34]. The most common sources of funding were non-private sources (*n* = 10, 59%) such as government grants and non-profit organisations, but some also received funding from private sources (*n* = 3, 18%).

In terms of study strategies, there was a dominance of studies using a hypothetical cohort (*n* = 15, 88%) with only two studies using data from a RCT [38, 42]. A range of perspectives were used to evaluate the cost-effectiveness of PGx-guided prescribing. The most common perspective was the societal perspective (*n* = 6, 35%), including both direct and indirect costs, and most of the remaining studies used perspectives that only consider direct costs (*n* = 9, 53%). However, one of these studies from the Canadian healthcare system perspective, also included indirect costs such as rent and employment income assistance, which are more aligned with the societal perspective [41]. Two studies also explored two perspectives (12%) [33, 42]. Time horizons varied significantly with the most common being between three and ten years (*n* = 7, 41%), and a lifetime horizon was only adopted by two studies (12%) [26, 32]. Eight studies (47%) also comprehensively explored uncertainty by carrying out deterministic, probabilistic, and scenario analyses.

Quality appraisal using the QHES instrument resulted in all studies achieving a high-quality score, with an average score of 85.41 (range 75–97; Supplementary Table 7). Strengths included clearly stated objectives, appropriate handling of uncertainty, use of valid health outcomes and well justified recommendations/conclusions. Common limitations included limited reporting of the reason for choosing the study perspective, not covering each of the major short-term, long-term and negative outcomes in their primary outcome(s), and frequent use of short time horizons which may not fully capture long term outcomes.

The certainty of the evidence in this review is moderate. There is a potential risk of bias with several studies declaring conflicts of interest, only half of the studies comprehensively exploring uncertainty and external validation of the model was often unclear. Indirectness of results was minimal, although one study focused on an elderly cohort ( ≥ 60 years old) on nortriptyline, limiting the generalisability of their results [27]. While 15 of 17 studies favoured PGx-guided prescribing, ICER results were inconsistent (dominant to $3.93 million). Imprecision was a concern due to the lack of uncertainty measures reported for cost and QALY outcomes. There was also no significant publication bias present (Supplementary Tables 15–17).

Summarising the study findings revealed a range of conclusions on the cost-effectiveness of PGx-guided prescribing for psychiatric conditions (Table 1). All studies modeled PGx-guided prescribing as a discrete intervention compared to standard care and did not explicitly incorporate real-world implementation barriers such as capacity and structural constraints. Despite this, eight studies (47%) found implementing PGx-led prescribing to be cost-effective, or likely to be depending on the WTP threshold, 41% (*n* = 7) found it to be the dominant strategy, with only one study finding it to be not cost-effective (6%), and one study reporting inconclusive results (6%). However, the extent of cost-effectiveness varied considerably. Two studies in particular reported much higher ICER values. Berm and colleagues reported an ICER of 1.3 million EUR/QALY gained and concluded PGx-led prescribing was not cost-effective, but interestingly Girardin and colleagues concluded PGx-guided prescribing was cost-effective despite having an ICER value of 3.93 million USD/QALY gained [27, 30]. Girardin and colleagues reported their ICER using the genotype-guided strategy as the reference, rather than standard care. For consistency, their results were recalculated with standard care as the reference, resulting in reversal of the signs of the incremental cost and effects but did not change the ICER value or the overall cost-effectiveness conclusion. Full data extraction tables available in Supplementary Tables 8-13.

**Table 1:** Study findings.

Study	Condition	PGx Test	ICER (Cost/QALY gained)^b^	WTP Threshold (Cost/QALY gained)	Study Conclusion^d^	Funding
ter Hark (2025) [42]	MDD	CYP2C19 and CYP2D6	26 weeks healthcare: −434 166 (EUR)	50 000 (EUR)	Dominant & cost-saving	Non-private
Skokou (2024) [38]^,a^	MDD	CYP2C19 or CYP2D6	−122 400 (EUR)	Not clear	Cost-effective	Non-private
ter Hark (2025) [42]	MDD	CYP2C19 and CYP2D6	13 weeks societal: −104 000 (EUR)	50 000 (EUR)	Not cost-effective	Non-private
Groessl (2018) [31]	MDD	IDgenetix Test	Sev MDD: −34 176 (USD)	50 000 (USD)	Dominant & cost-saving	Private
Groessl (2018) [31]	MDD	IDgenetix Test	Mod-Sev MDD: −25 980 (USD)	50 000 (USD)	Dominant & cost-saving	Private
Tanner (2020) [41]	DEP	GeneSight Panel	−14 454 (CAD)	50 000 (CAD)	Dominant & cost-saving	Private and non-private
Ghanbarian (2023) [29]	MDD	CYP2C19 and CYP2D6	−12 929 (CAD)	50 000 (CAD)	Dominant & cost-saving	Non-private
Hornberger (2015) [32]	MDD	CYP2D6, CYP2C19, CYP2C9, CYP1A2, SLC6A4 and HTR2A	−11 911 (USD)	50 000 (USD)	Dominant & cost-saving	Private
Lopez-Saavedra (2024a) [33]	MDD	VIPOA Panel	Societal: −7 820.56 (EUR)	21 000 (EUR)	Dominant & cost-saving	No Funding
Lopez-Saavedra (2024b) [33]	MDD	VIPOA Panel	Healthcare provider: −1 130.16 (EUR)	21 000 (EUR)	Dominant & cost-saving	No Funding
Najafzadeh (2017) [36]	DEP and/or ANX	IDgenetix Test	−3 567 (USD)	50 000 (USD)	Dominant & cost-saving	No Funding
Abushanab (2024) [26]	MDD	CYP2C19 and CYP2D6	−757 (QAR) (−208 USD)	150 000 (USD)	Dominant & cost-saving	No statement
ter Hark (2025) [42]	MDD	CYP2C19 and CYP2D6	13 weeks healthcare: 11 840 (EUR)	50 000 (EUR)	Not cost-effective	Non-private
Sluiter (2018) [39]	AUD	OPRM1	13 350 (EUR)	80 000 (EUR)	Likely to be cost-effective	No statement
Ninomiya (2022) [35]	SCZ	HLA-B, HLA-DQB1, and SLCO1B3-SLCO1B7	16 215 (GBP)	30 000 (GBP)	Likely to be cost-effective	Non-private
Rejon-Parilla (2014) [37]	SCZ	CYP2D6	19 252 (GBP)	20 000–30 000 (GBP)^c^	Likely to be cost-effective	No Funding
Ninomiya (2021a) [34]	SCZ	Japan: HLA-B	21 024 (GBP)	37 651 (GBP)	Likely to be cost-effective	Non-private
Ninomiya (2021b) [34]	SCZ	UK: HLA-B and HLA-DQB1	21 343 (GBP)	30 000 (GBP)	Likely to be cost-effective	Non-private
Carta (2022a) [28]	MDD	CYP2D6	46 908 (EUR)	75 000 (EUR)	Likely to be cost-effective	Non-private
Carta (2022b) [28]	MDD	CYP2C19	60 094 (EUR)	75 000 (EUR)	Likely to be cost-effective	Non-private
Sluiter (2019) [40]	MDD	CYP2D6	77 406 (EUR)	80 000 (EUR)	Likely to be cost-effective	Private
Berm (2016) [27]	MDD	CYP2D6	1 333 148 (EUR)	50 000 (EUR)	Not cost-effective	Non-private
ter Hark (2025) [42]	MDD	CYP2C19 and CYP2D6	26 weeks societal: 1 420 000 (EUR)	50 000 (EUR)	Not cost-effective	Non-private
Girardin (2019) [30]	SCZ	HLA-B and HLA-DQB1	3 930 000 (USD)	Not clear	Cost-effective	Non-private

A summary of the quantitative findings of all 17 studies in this review sorted by the ICER values. The table also includes the psychiatric condition of interest, the PGx test used, WTP threshold, and funding source to provide context to the conclusions.

*DEP* Depression, *ANX* Anxiety, *MDD* Major Depressive Disorder, *Mod-Sev MDD* Moderate to Severe Major Depressive Disorder, *SCZ* Schizophrenia, *AUD* Alcohol Use Disorder, *PGx* Pharmacogenetic, *VIPOA* Very Important Pharmacogene Open Array, *ICER* Incremental Cost-Effectiveness Ratio, *QALY* Quality-Adjusted Life-Year, *UK* United Kingdom, *WTP* Willingness-to-Pay Threshold

^a^ Skokou and colleagues looked at multiple psychiatric conditions, however, the cohorts of schizophrenia and bipolar disorder were excluded from their conclusions due to inaccurate self-reported quality of life and so are not included here [38].

^b^ Any shaded ICER results were not provided by the studies as they were negative ICERs, which are typically not reported due to the ambiguity in interpretation. These ICER values were calculated using data provided in the papers.

^c^ This study did not definitively state their WTP threshold but refers to the use of NICE guidelines to establish cost-effectiveness.

^d^ Study conclusion as reported by the authors of the economic evaluations.

**Fig. 2:**
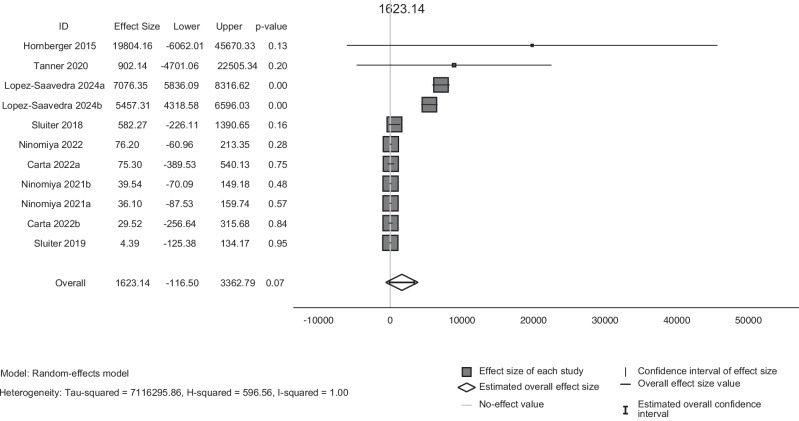
Forest plot on the cost-effectiveness of pharmacogenomics-guided prescribing for psychiatric disorders across all study groups. This forest plot shows the effect sizes (Incremental Net Benefit) from a meta-analysis of pharmacogenomics-guided prescribing compared to standard care. In some cases, multiple study groups were derived from a single study: Carta (2022a) focuses on pharmacogenetic-testing of CYP2D6, while Carta (2022b) examines CYP2C19, Lopez-Saavedra (2024a) evaluates a societal perspective, whereas Lopez-Saavedra (2024b) uses a healthcare provider perspective, Ninomiya (2021a) is simulating a Japanese population, and Ninomiya (2021b) is simulating a UK population [28, 33, 34]. Heterogeneity was assessed using various tests including I^2^, H^2^ and τ^2^.

Following the narrative synthesis, we then conducted a meta-analysis using data extracted from CE planes (Supplementary Table 14). A random-effects model was used to pool the INBs of the total 11 study groups, all of which were positive, resulting in a pooled INB of £1 623.14 (95% Confidence Intervals (CI): -£116.50 to £3 362.79) (Fig. 2). However, this pooled effect was non-significant (*p* = 0.07) at a 95% confidence level. The study weightings were similar across all study groups (ranging from 10.48% to 11.07%), apart from two studies. These two studies had the largest 95% CIs and therefore the smallest weights in the overall pooled INB (0.43% and 1.42%) [32, 41]. All heterogeneity tests also indicated that there was substantial heterogeneity present (I^2^ = 100%, Tau^2^ = 7 116 295.86 and Cochran’s Q = 215.61, df = 10, *p* = 0.00).

**Fig. 3:**
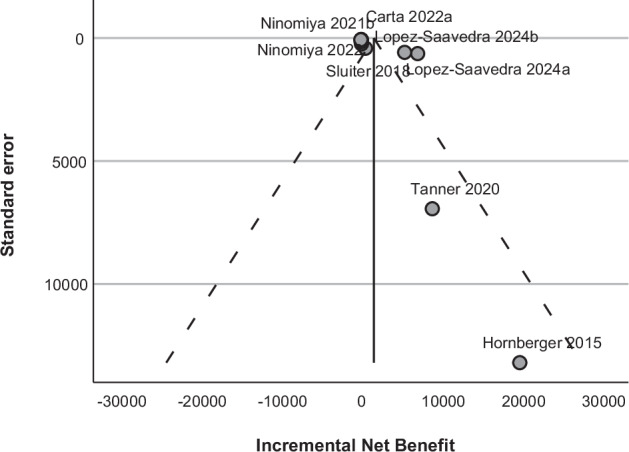
Funnel plot for meta-analysis of total study groups. Funnel plot to assess publication for total 11 study groups. The dots represent primary studies dashed lines indicate the 95% pseudo confidence intervals and the solid line is the estimated overall effect size. The funnel plot shows visual asymmetry with the study by Tanner and colleagues and the study by Hornberger and colleagues separating from other studies, and the study groups by Lopez-Saavedra and colleagues also fall just outside the 95% pseudo-confidence intervals [32, 33, 41].

**Fig. 4:**
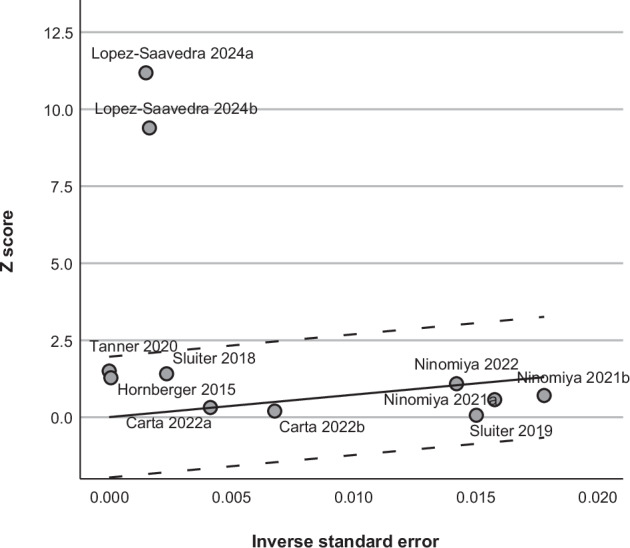
Galbraith plot for meta-analysis of total study groups. Galbraith plot assessing heterogeneity for total 11 study groups. The dots represent primary studies dashed lines indicate the 95% confidence interval region and the solid line is the regression line. The Galbraith plot highlights study groups by Lopez-Saavedra and colleagues as potential outliers [33].

Given the substantial heterogeneity, funnel and Galbraith plots were used to assess heterogeneity and publication bias (Figs. 3 and 4). The funnel plot showed visual asymmetry with two studies, which typically indicates publication bias, however the Egger’s regression-based test showed no significant evidence of publication bias (intercept *p* = 0.27)(Supplementary Table 15). Additionally, the two study groups by Lopez-Saavedra and colleagues fell just outside the 95% pseudo-confidence intervals on the funnel plot and were also outliers on the Galbraith plot [33]. A leave-one-out analysis (Table 2) revealed that removing these study groups by Lopez-Saavedra and colleagues, affected τ², H², and Cochran’s Q statistic the most, indicating their contribution to the overall heterogeneity. A meta-regression was not performed due to the small number of studies and subgroup analyses were only exploratory due to the significant heterogeneity present (Supplementary Figures 1–4).

**Table 2:** Heterogeneity and homogeneity measures from leave-one-out analysis.

	Heterogeneity measures following study removal			Test of homogeneity following study removal		
Excluded Study	τ²	H^2^	I^2^ (%)	Cochran’s Q statistic	Degrees of Freedom	Significance
Carta (2022a) [28]	7 913 899.030	717.960	99.9	215.612	9	<0.01
Carta (2022b) [28]	7 893 102.325	686.032	99.9	215.520	9	<0.01
Hornberger (2015) [32]	7 055 099.245	657.034	99.8	213.377	9	<0.01
Lopez-Saavedra (2024a) [33]	3 106 361.215	288.800	99.7	92.836	9	<0.01
Lopez-Saavedra (2024b) [33]	5 533 707.784	513.342	99.8	129.483	9	<0.01
Ninomiya (2021a) [34]	7 896 705.953	517.172	99.8	215.171	9	<0.01
Ninomiya (2021b) [34]	7 898 394.310	483.488	99.8	215.110	9	<0.01
Ninomiya (2022) [35]	7 915 871.059	549.630	99.8	215.609	9	<0.01
Sluiter (2018) [39]	8 095 071.892	747.292	99.9	214.078	9	<0.01
Sluiter (2019) [40]	7 881 119.857	530.759	99.8	214.259	9	<0.01
Tanner (2020) [41]	7 088 380.081	660.115	99.8	213.994	9	<0.01
No studies excluded	7 116 295.855	596.557	99.8	215.612	10	<0.01

Heterogeneity and homogeneity measures (τ², H², I² and Cochran’s Q test) following the removal of each study in the leave-one-out analysis.

In attempt to explore if there was a subgroup of studies that were not statistically heterogeneous, the two study groups by Lopez-Saavedra and colleagues were excluded [33]. The study by Hornberger and colleagues was also excluded due to a methodological difference, as this was the only study with a lifetime horizon [32].

The meta-analysis for the homogenous subgroup of studies produced a positive INB (£41.54, 95% CI -£18.27 to £101.35) however, the overall observed effect remained non-significant (*p* = 0.17) (Supplementary Figure 5). As there was no substantial heterogeneity (I^2^ = 0%), a fixed-effect model was used for this meta-analysis. Results using a random-effects model are also included in Supplementary Figure 6 and funnel and Galbraith plots following study removal in Supplementary Figure 7.

## Discussion

Our findings suggest PGx-guided prescribing has the potential to be cost-effective compared to standard care in psychiatry, although the results varied across study contexts. Multi-gene testing for depression appears particularly promising with each model-based study evaluating this approach finding it to be cost-saving [26, 29, 31–33, 36, 41]. These studies evaluated PGx prescribing recommendations for at least the CYP2C19 and CYP2D6 genes. A recent study exploring the impact of PGx-guided prescribing on the occurrence of ADRs found that 9% of all ADRs in the UK between 1963 and 2024, were caused by PGx actionable drugs, 75% of which were metabolised by CYP2D6, CYP2C19, and SLCO1B1 [43]. They further reported that ∼4% of these PGx-actionable ADRs were due to psychiatric medications, most of which were severe or fatal. Given that ADRs have been estimated to cost >£2.2 billion annually in the UK, it is not surprising that implementing PGx-prescribing in psychiatry, including at least these two genes, could result in cost-savings [44]. While these results are promising, four of these studies evaluated multi-gene panels and were either industry-funded or disclosed a conflict of interest, raising queries about the rigour and reproducibility of the outcomes [31, 32, 36, 41].

Evaluations of multi-gene PGx-guided prescribing for schizophrenia exclusively looked at the antipsychotic clozapine [30, 34, 35]. Clozapine is currently the gold standard treatment for treatment-resistant schizophrenia; however, its use has been found to cause clozapine-induced agranulocytosis/granulocytopenia (CIAG), a rare but life-threatening ADR [45–48]. For patient safety, the current standard of care involves routine haematological monitoring, which is included in 90% of guidelines across 102 countries and mandatory in 45% [49]. This monitoring has been found to account for 70% of the total treatment costs for clozapine, demonstrating its economic burden [50]. The model-based evaluations in this review evaluated the use of a PGx test to identify individuals at increased risk of CIAG and to stratify the monitoring strategy accordingly. Their findings suggest that reducing monitoring in low-risk patients and increasing surveillance for those with increased risk, could be a cost-effective approach compared to the current standard of care.

Single-gene testing was also generally cost-effective across the model-based studies, with only one study reporting it to be not cost-effective [27]. The exception focused on implementing PGx-informed prescribing for CYP2D6 in elderly depressed Dutch patients treated with nortriptyline. Cost-effectiveness in their analysis was mainly dependent on genotyping costs however they found when the cost was reduced to <€35 per test, PGx-guided prescribing was dominant. While the cost of PGx-led prescribing likely impacts cost-effectiveness, several studies in this review found PGx-informed prescribing to be cost-effective, or even cost-saving, despite having PGx test costs substantially higher than the cost used by Berm and colleagues [27, 31, 32, 36, 41]. This suggests that there are other methodological and contextual factors such as test scope (single vs multi-gene), the patient population and the healthcare setting that are also important determinants of cost-effectiveness. Such clinical and methodological heterogeneity is a common challenge in systematic reviews of economic evidence and makes pooling ICERs or INBs difficult. As a result, there is currently no universally accepted framework for the synthesis of economic evidence, and a narrative synthesis is often the preferred method [51]. While a narrative synthesis is informative, it lacks the precision of a pooled effect which can be beneficial for informing policy.

Trial-based economic evaluations of PGx-guided prescribing remain limited, with only two of the studies in this review adopting this approach [38, 42]. While trial-based evaluations offer certain advantages such as using direct patient-level data, they also have methodological limitations, such as the typical use of short time horizons [52, 53]. For example, ter Hark and colleagues found CYP2D6 and CYP2C19-guided tricyclic antidepressant prescribing to be cost-saving at 26 weeks from the healthcare perspective, but not cost-effective at 13 weeks or from a societal perspective [42]. NICE recommends using a time horizon that is long enough to capture all relevant costs and outcomes [54]. Considering MDD has been found to be unremitting in 15% of cases and recurrent in 35% cases over long-term follow-up, these short time horizons may underestimate the value of PGx-guided prescribing by missing long-term health and societal impacts [55]. Future trial-based economic evaluations require longer follow-up periods for a more comprehensive real-world evaluation.

Our meta-analysis of the model-based studies showed that there were four study groups that had effect sizes much larger than those of the remaining studies [32, 33, 41]. Factors that may have contributed to these larger effect sizes include that these studies were evaluating multi-gene testing, methodological differences such as employing a Markov microsimulation modelling approach, using a lifetime horizon, and being privately funded [32, 33, 41]. Two of these studies also had considerable standard errors (13 197.27 and 6 940.54) which may reflect the fact that these were the only studies to carry out their own meta-analyses to inform their response or remission rates for their model [32, 41]. Meta-analyses are considered the most reliable source of evidence, however the economic evaluation by Hornberger and colleagues only included one RCT in their meta-analysis and Tanner and colleagues included two [32, 41]. The resulting relative benefit ratios produced for the PGx cohort compared to standard care had limited precision, ranging from 1.17 to 2.49 and from 1.15 to 1.91 respectively. The small number and quality of studies included in their meta-analyses may have contributed to the observed uncertainty in their results.

Overall, the findings of this review are consistent with previous literature in the field. Karamperis and colleagues were the first to evaluate the cost-effectiveness of PGx-guided prescribing in psychiatry, using a narrative synthesis, and found that 89% were in favour of PGx-informed prescribing, 50% of which were cost-effective, and 39% found to be dominant [15]. A recent systematic review looking at PGx-led prescribing of antipsychotics found that all studies included were either in favour of PGx-guided prescribing or there was no difference compared to standard care, for both clinical and economic outcomes [56]. While not specifically looking at psychiatric medications, another review found that 82% of their studies evaluating antidepressants favoured PGx-guided prescribing [57]. However, some reviews were unable to come to a conclusion on cost-effectiveness due to a lack of consistency in economic modelling methods, variability in the outcomes that were reported and the lack of replicated findings to support cost-effectiveness [58, 59]. These limitations highlight the need for increased consistency in economic modelling methods and reporting of results to ensure more confident conclusions.

This systematic review and meta-analysis has several limitations which should be considered when interpreting the results. Firstly, excluding studies using a pre-post study design or comparing congruent versus incongruent prescribing may have limited the scope of the review. These studies, while informative, may have introduced confounding factors by not having separate cohorts, making economic comparisons more complex. Secondly, the use of the QHES instrument, a subjective, knowledge-based tool, with dichotomous scoring may have been a limitation. The QHES instrument was not designed to assess economic evaluations of PGx interventions which present specific methodological challenges [60]. However, the QHES instrument has been used in previous systematic reviews evaluating the cost-effectiveness of PGx-guided prescribing [15, 47]. Thirdly, currency conversions were calculated using the ‘CCEMG-EPPI-Centre Cost Converter’ tool which relies on International Money Fund staff estimates, which may have introduced uncertainty, however, provided a standardised approach for currency conversion. Fourthly, restricting the review to recent studies could be a limitation, however this was decided due to the rapidly changing nature of factors such as inflation rates. Finally, the fact that our meta-analysis had a small number of studies also reduces the confidence of our findings and so an update of the literature could hopefully produce more conclusive results in the future.

The findings from this systematic review and meta-analysis suggest that PGx-informed prescribing, particularly using a multi-gene testing approach for depressed patients, may be cost-effective compared to standard care and potentially even cost-saving. However, methodological challenges, such as the presence of substantial heterogeneity, limit the robustness of conclusions and highlight the need for more consistent approaches in future economic evaluations. Future evaluations should also consider evaluating the cost-effectiveness for a broader range of antipsychotics, rather than just clozapine, and where possible use real-world data from long-term RCTs to better reflect real-world outcomes. Addressing these gaps could produce more robust conclusions on the cost-effectiveness of PGx-guided prescribing for psychiatric medications in the future.

## Supplementary information

Supplementary materials

## Data Availability

All data extracted during this systematic review are available in the manuscript or in the supplementary material. Data extracted from CE planes are available at: https://github.com/Ellenm10/Systematic-Review/tree/main.
