# Supplementary material for: Economic effectiveness of pharmacogenomics-guided prescribing for psychiatric disorders: a systematic review and meta-analysis
Source: Pharmacogenomics J. 2026 Apr 4;26(2):12. doi: 10.1038/s41397-026-00408-2 (PMC13050323; doi:10.1038/s41397-026-00408-2)
Supplement: Supplementary file 1 — Supplementary materials [file 41397_2026_408_MOESM1_ESM.docx]

**Economic Effectiveness of Pharmacogenomics-Guided Prescribing for Psychiatric Disorders: A Systematic Review and Meta-Analysis**

**Running Title:** Economic Value of Pharmacogenomics in Psychiatry

Ellen R Mason, BSc^1^ , Mohamed Y Ali, MRes^1^, David S Gibson, PhD^1^, Elaine K Murray, PhD^1^, Richéal M Burns, PhD^2,3^, Catriona Kelly, PhD^1^

^1^ Personalised Medicine Centre, School of Medicine, Ulster University, C-TRIC Builiding, Altanagelvin Hospital Campus, Glenshane Road, Derry⁓Londonderry, BT47 6SB, UK

^2^ Department of Health and Nutritional Science, Atlantic Technological University, Faculty of Science, Sligo, Ireland, F91 YW50

^3^ Health and Biomedical Research Centre (HEAL), Atlantic Technological University, Faculty of Science, Sligo, Ireland, F91 YW50

**Correspondence to:** Dr Catriona Kelly

Personalised Medicine Centre, Ulster University, C-TRIC Building, Altnagelvin Hospital, Derry~Londonderry, BT47 6SB

Email: [c.kelly@ulster.ac.uk](mailto:c.kelly@ulster.ac.uk)

**Supplementary Material**

Contents

[Supplementary Tables 3](#_Toc206504970)

[Supplementary Table 1: MEDLINE and Embase Search strategy 3](#_Toc206504971)

[Supplementary Table 2: PsycINFO Search strategy 4](#_Toc206504972)

[Supplementary Table 3: PRISMA 2020 abstract checklist 5](#_Toc206504973)

[Supplementary Table 4: PRISMA 2020 checklist 6](#_Toc206504974)

[Supplementary Table 5: QHES questions and adaptions 9](#_Toc206504975)

[Supplementary Table 6: Excluded studies and reasons for exclusion 10](#_Toc206504976)

[Supplementary Table 7: QHES results 11](#_Toc206504977)

[Supplementary Table 8: Data extraction of article information 12](#_Toc206504978)

[Supplementary Table 9: Data extraction of study characteristics 13](#_Toc206504979)

[Supplementary Table 10: Data extraction of study methods 15](#_Toc206504980)

[Supplementary Table 11: Data extraction of study uncertainty assessments, willingness-to-pay thresholds, and outcomes 17](#_Toc206504981)

[Supplementary Table 12: Data extraction of patients and intervention information 18](#_Toc206504982)

[Supplementary Table 13: Summary estimates extracted from full-text papers 20](#_Toc206504983)

[Supplementary Table 14: Data used to calculate incremental net benefit and its variance 22](#_Toc206504984)

[Supplementary Table 15: Egger’s regression-based test for total study groups 23](#_Toc206504985)

[Supplementary Table 16: Egger’s regression-based test for homogeneous subgroup using fixed-effects model 24](#_Toc206504986)

[Supplementary Table 17: Egger’s regression-based test for homogeneous subgroup using random-effects model 25](#_Toc206504987)

[Supplementary Figures 26](#_Toc206504988)

[Supplementary Figure 1: Subgroup analysis by testing approach 26](#_Toc206504989)

[Supplementary Figure 2: Subgroup analysis by psychiatric disorder 27](#_Toc206504990)

[Supplementary Figure 3: Subgroup analysis by time horizon 28](#_Toc206504991)

[Supplementary Figure 4: Subgroup analysis by willingness-to-pay threshold 29](#_Toc206504992)

[Supplementary Figure 5: Forest plot on the cost-effectiveness of pharmacogenomics-guided prescribing for psychiatric disorders for a homogenous subgroup of studies using a fixed-effects model 30](#_Toc206504993)

[Supplementary Figure 6: Forest plot on the cost-effectiveness of pharmacogenomics-guided prescribing for psychiatric disorders for a homogenous subgroup of studies using a random-effects model 31](#_Toc206504995)

[Supplementary Figure 7: Funnel and Galbraith plots for statistically homogenous meta-analysis 32](#_Toc206504996)

[Supplementary Methods 33](#_Toc206504997)

[Review protocol 33](#_Toc206504998)

[References 37](#_Toc206504999)

# Supplementary Tables

## Supplementary Table 1: MEDLINE and Embase Search strategy

| **Search strategy** – MEDLINE | |
| --- | --- |
| **Search #** | **Searches** |
| 1 | exp Mental Disorders/ |
| 2 | (mental* or psychiatr* or depressi* or schizo* or psycho* or bipolar* or anxi* or obsess* or post-traumat* or dissociative*) |
| 3 | 1 or 2 |
| 4 | Pharmacogenetics/ or Pharmacogenomic Testing/ or Cytochrome P-450 Enzyme System/ or Cytochrome P450 Family 1/ or Cytochrome P450 Family 2/ or Cytochrome P450 Family 3/ |
| 5 | pharmacogen** or cytochrome* or CYP2D6 or CYP2C19 or CYP2C9 or CYP2B6 or CYP3A4 or CYP1A2 |
| 6 | 4 or 5 |
| 7 | "Costs and Cost Analysis"/ or Cost-Benefit Analysis/ or Cost-Effectiveness Analysis/ |
| 8 | economic* or cost** or "incremental cost-effectiveness ratio*" or "cost-utility analysis" or "health economic evaluation" |
| 9 | 7 or 8 |
| 10 | 3 and 6 and 9 |
| 11 | limit 10 to (English language and "remove preprint records" and yr="2014 - 2025") |

## Supplementary Table 2: PsycINFO Search strategy

| **Search strategy** – PsycINFO | |
| --- | --- |
| **Search #** | **Searches** |
| 1 | exp Mental Disorders/ |
| 2 | (mental* or psychiatr* or depressi* or schizo* or psycho* or bipolar* or anxi* or obsess* or post-traumat* or dissociative*) |
| 3 | 1 or 2 |
| 4 | Pharmacogenetics/ |
| 5 | pharmacogen** or cytochrome* or CYP2D6 or CYP2C19 or CYP2C9 or CYP2B6 or CYP3A4 or CYP1A2 |
| 6 | 4 or 5 |
| 7 | "Costs and Cost Analysis"/ |
| 8 | economic* or cost** or "incremental cost-effectiveness ratio*" or "cost-utility analysis" or "health economic evaluation" |
| 9 | 7 or 8 |
| 10 | 3 and 6 and 9 |
| 11 | limit 10 to (peer reviewed journal and english language and yr="2014 - 2025") |

## Supplementary Table 3: PRISMA 2020 abstract checklist

| **Section and Topic** | **Item #** | **Checklist item** | **Reported (Yes/No)** |
| --- | --- | --- | --- |
| **TITLE** | | |  |
| Title | 1 | Identify the report as a systematic review. | Yes |
| **BACKGROUND** | | |  |
| Objectives | 2 | Provide an explicit statement of the main objective(s) or question(s) the review addresses. | Yes |
| **METHODS** | | |  |
| Eligibility criteria | 3 | Specify the inclusion and exclusion criteria for the review. | Yes |
| Information sources | 4 | Specify the information sources (e.g. databases, registers) used to identify studies and the date when each was last searched. | Yes |
| Risk of bias | 5 | Specify the methods used to assess risk of bias in the included studies. | Yes |
| Synthesis of results | 6 | Specify the methods used to present and synthesise results. | Yes |
| **RESULTS** | | |  |
| Included studies | 7 | Give the total number of included studies and participants and summarise relevant characteristics of studies. | Yes |
| Synthesis of results | 8 | Present results for main outcomes, preferably indicating the number of included studies and participants for each. If meta-analysis was done, report the summary estimate and confidence/credible interval. If comparing groups, indicate the direction of the effect (i.e. which group is favoured). | Yes |
| **DISCUSSION** | | |  |
| Limitations of evidence | 9 | Provide a brief summary of the limitations of the evidence included in the review (e.g. study risk of bias, inconsistency and imprecision). | Yes |
| Interpretation | 10 | Provide a general interpretation of the results and important implications. | Yes |
| **OTHER** | | |  |
| Funding | 11 | Specify the primary source of funding for the review. | Yes |
| Registration | 12 | Provide the register name and registration number. | Yes |

## Supplementary Table 4: PRISMA 2020 checklist

| **Section and Topic** | **Item #** | **Checklist item** | **Location where item is reported** |
| --- | --- | --- | --- |
| **TITLE** | | |  |
| Title | 1 | Identify the report as a systematic review. | Title |
| **ABSTRACT** | | |  |
| Abstract | 2 | See the PRISMA 2020 for Abstracts checklist. | Suppl. Table 3 |
| **INTRODUCTION** | | |  |
| Rationale | 3 | Describe the rationale for the review in the context of existing knowledge. | Page 4-5 |
| Objectives | 4 | Provide an explicit statement of the objective(s) or question(s) the review addresses. | Page 5 |
| **METHODS** | | |  |
| Eligibility criteria | 5 | Specify the inclusion and exclusion criteria for the review and how studies were grouped for the syntheses. | Suppl. Methods and Page 5 |
| Information sources | 6 | Specify all databases, registers, websites, organisations, reference lists and other sources searched or consulted to identify studies. Specify the date when each source was last searched or consulted. | Page 5 |
| Search strategy | 7 | Present the full search strategies for all databases, registers and websites, including any filters and limits used. | Suppl. Tables 1 and 2 |
| Selection process | 8 | Specify the methods used to decide whether a study met the inclusion criteria of the review, including how many reviewers screened each record and each report retrieved, whether they worked independently, and if applicable, details of automation tools used in the process. | Page 5-6 |
| Data collection process | 9 | Specify the methods used to collect data from reports, including how many reviewers collected data from each report, whether they worked independently, any processes for obtaining or confirming data from study investigators, and if applicable, details of automation tools used in the process. | Pages 6-7 |
| Data items | 10a | List and define all outcomes for which data were sought. Specify whether all results that were compatible with each outcome domain in each study were sought (e.g. for all measures, time points, analyses), and if not, the methods used to decide which results to collect. | Pages 6-7 |
|  | 10b | List and define all other variables for which data were sought (e.g. participant and intervention characteristics, funding sources). Describe any assumptions made about any missing or unclear information. | Pages 6-7 |
| Study risk of bias assessment | 11 | Specify the methods used to assess risk of bias in the included studies, including details of the tool(s) used, how many reviewers assessed each study and whether they worked independently, and if applicable, details of automation tools used in the process. | Page 8 |
| Effect measures | 12 | Specify for each outcome the effect measure(s) (e.g. risk ratio, mean difference) used in the synthesis or presentation of results. | Page 6-7 |
| Synthesis methods | 13a | Describe the processes used to decide which studies were eligible for each synthesis (e.g. tabulating the study intervention characteristics and comparing against the planned groups for each synthesis (item #5)). | Pages 5-6 |
|  | 13b | Describe any methods required to prepare the data for presentation or synthesis, such as handling of missing summary statistics, or data conversions. | Page 7-8 |
|  | 13c | Describe any methods used to tabulate or visually display results of individual studies and syntheses. | Pages 6 and 8 |
|  | 13d | Describe any methods used to synthesize results and provide a rationale for the choice(s). If meta-analysis was performed, describe the model(s), method(s) to identify the presence and extent of statistical heterogeneity, and software package(s) used. | Pages 7-8 |
|  | 13e | Describe any methods used to explore possible causes of heterogeneity among study results (e.g. subgroup analysis, meta-regression). | Page 8 |
|  | 13f | Describe any sensitivity analyses conducted to assess robustness of the synthesized results. | Page 8 |
| Reporting bias assessment | 14 | Describe any methods used to assess risk of bias due to missing results in a synthesis (arising from reporting biases). | Page 8 |
| Certainty assessment | 15 | Describe any methods used to assess certainty (or confidence) in the body of evidence for an outcome. | Page 8 |
| **RESULTS** | | |  |
| Study selection | 16a | Describe the results of the search and selection process, from the number of records identified in the search to the number of studies included in the review, ideally using a flow diagram. | Page 9 and Fig.1 |
|  | 16b | Cite studies that might appear to meet the inclusion criteria, but which were excluded, and explain why they were excluded. | Suppl. Table 6 |
| Study characteristics | 17 | Cite each included study and present its characteristics. | Pages 9-10 and Table 1 |
| Risk of bias in studies | 18 | Present assessments of risk of bias for each included study. | Suppl. Tables 5, 15-17, Suppl. Fig. 7, Fig. 3-4 and Pages 10-11 |
| Results of individual studies | 19 | For all outcomes, present, for each study: (a) summary statistics for each group (where appropriate) and (b) an effect estimate and its precision (e.g. confidence/credible interval), ideally using structured tables or plots. | Suppl. Table 13 |
| Results of syntheses | 20a | For each synthesis, briefly summarise the characteristics and risk of bias among contributing studies. | Table 1 and Suppl. Table 7 and 15 |
|  | 20b | Present results of all statistical syntheses conducted. If meta-analysis was done, present for each the summary estimate and its precision (e.g. confidence/credible interval) and measures of statistical heterogeneity. If comparing groups, describe the direction of the effect. | Pages 10-11, Fig. 2 and Suppl. Table 14 |
|  | 20c | Present results of all investigations of possible causes of heterogeneity among study results. | Pages 10-11, Fig. 2, and Suppl. Figs 1-4 |
|  | 20d | Present results of all sensitivity analyses conducted to assess the robustness of the synthesized results. | Table 2 |
| Reporting biases | 21 | Present assessments of risk of bias due to missing results (arising from reporting biases) for each synthesis assessed. | Pages 12-13, Fig. 3, Suppl. Tables 15-17 |
| Certainty of evidence | 22 | Present assessments of certainty (or confidence) in the body of evidence for each outcome assessed. | Pages 10-11 |
| **DISCUSSION** | | |  |
| Discussion | 23a | Provide a general interpretation of the results in the context of other evidence. | Pages 16-17 |
|  | 23b | Discuss any limitations of the evidence included in the review. | Page 17-18 |
|  | 23c | Discuss any limitations of the review processes used. | Page 17-18 |
|  | 23d | Discuss implications of the results for practice, policy, and future research. | Pages 18 |
| **OTHER INFORMATION** | | |  |
| Registration and protocol | 24a | Provide registration information for the review, including register name and registration number, or state that the review was not registered. | Page 6 |
|  | 24b | Indicate where the review protocol can be accessed, or state that a protocol was not prepared. | Page 6, Suppl. Methods |
|  | 24c | Describe and explain any amendments to information provided at registration or in the protocol. | N/A |
| Support | 25 | Describe sources of financial or non-financial support for the review, and the role of the funders or sponsors in the review. | Page 18 |
| Competing interests | 26 | Declare any competing interests of review authors. | Page 18 |
| Availability of data, code and other materials | 27 | Report which of the following are publicly available and where they can be found: template data collection forms; data extracted from included studies; data used for all analyses; analytic code; any other materials used in the review. | Availability of data statement |

*From:*  Page MJ, McKenzie JE, Bossuyt PM, Boutron I, Hoffmann TC, Mulrow CD, et al The PRISMA 2020 statement: an updated guideline for reporting systematic reviews. BMJ 2021;372:n71. doi: 10.1136/bmj.n71. This work is licensed under CC BY 4.0. To view a copy of this license, visit <https://creativecommons.org/licenses/by/4.0/>

## Supplementary Table 5: QHES questions and adaptions

| **#** | **Questions** |
| --- | --- |
|  |  |
| 1 | Was the study objective presented in a clear, specific, and measurable manner? |
| 2 | Were the perspective of the analysis (societal, third-party payer, etc.) and reasons for its selection stated? |
| 3 | Were variable estimates used in the analysis from the best available source (i.e., randomized control trial - best, expert opinion - worst)? |
| 4 | If estimates came from a subgroup analysis, were the groups pre-specified at the beginning of the study? |
| 5 | Was uncertainty handled by (1) statistical analysis to address random events, (2) sensitivity analysis to cover a range of assumptions? |
| 6 | Was incremental analysis performed between alternatives for resources and costs? |
| 7 | Was the methodology for data abstraction (including the value of health states and other benefits) stated? |
| 8 | Did the analytic horizon allow time for all relevant and important outcomes? Were benefits and costs that went beyond 1 year discounted (3% to 5%) and justification given for the discount rate? |
| 9 | Was the measurement of costs appropriate and the methodology for the estimation of quantities and unit costs clearly described? |
| 10 | Were the primary outcome measure(s) for the economic evaluation clearly stated and did they include the major short-term, long-term and negative outcomes? |
| 11 | Were the health outcomes measures/scales valid and reliable? If previously tested valid and reliable measures were not available, was justification given for the measures/scales used? |
| 12 | Were the economic model (including structure), study methods and analysis, and the components of the numerator and denominator displayed in a clear, transparent manner? |
| 13 | Were the choice of economic model, main assumptions, and limitations of the study stated and justified? |
| 14 | Did the author(s) explicitly discuss direction and magnitude of potential biases? |
| 15 | Were the conclusions/recommendations of the study justified and based on the study results? |
| 16 | Was there a statement disclosing the source of funding for the study? |

Some of the QHES checklist questions are particularly relevant for model-based economic studies. For any studies in this systematic review that were not model-based but had the relevant economic and health-related outcomes, some questions were adapted accordingly.

- Question 7 to “was the methodology for data collection and measurement (including outcome measures and other relevant variables) clearly stated?”
- Question 12 to “were the study methods, analysis procedures and outcome measures displayed in a clear, transparent manner?”
- Question 13 to “were the choice of study design, main assumptions and limitations of the study clearly stated and justified?”

## Supplementary Table 6: Excluded studies and reasons for exclusion

| **Study** | **Reason for exclusion** | **Journal** |
| --- | --- | --- |
| Allen (2015)(1) | Conference abstract | CNS Spectrums |
| Basu (2024)(2) | Study protocol | PloS one |
| Benitez (2018)(3) | Lacked health and/or cost outcomes required for ICER calculation | Personalized medicine |
| Brown (2017)(4) | No distinct standard care cohort (congruent vs incongruent prescribing) | Clinical therapeutics |
| Callegari (2019)(5) | No distinct standard care cohort (pre-post design) and lacked health and/or cost outcomes required for ICER calculation | Journal of personalized medicine |
| Carrascal-Laso (2021)(6) | No distinct standard care cohort (pre-post design) and lacked health and/or cost outcomes required for ICER calculation | Pharmacogenomics and personalized medicine |
| Chatziandreou (2022)(7) | Conference abstract | Value in Health |
| Conley (2020)(8) | No distinct standard care cohort (Pre-post design) and lacked health and/or cost outcomes required for ICER calculation | Journal of the American Psychiatric Nurses Association |
| Edwards (2020)(9) | Conference abstract | CNS Spectrums |
| Espadaler (2017)(10) | Conference abstract | Value in Health |
| Fabbri (2021)(11) | Investigated the combination of PGx and clinical factors compared to clinical factors alone to guide combined pharmacotherapy and psychotherapy vs pharmacotherapy | Journal of affective disorders |
| Fagerness (2014)(12) | Lacked health and/or cost outcomes required for ICER calculation | The American journal of managed care |
| Jablonski (2020)(13) | No distinct standard care cohort (congruent vs incongruent prescribing) | Journal of geriatric psychiatry and neurology |
| Maciel (2018)(14) | Lacked health and/or cost outcomes required for ICER calculation | Neuropsychiatric disease and treatment |
| Mayhew (2017)(15) | Conference abstract | American Journal of Geriatric Psychiatry |
| Olson (2017)(16) | Lacked health and/or cost outcomes required for ICER calculation | The primary care companion for CNS disorders |
| Oslin (2021)(17) | Study protocol | Contemporary clinical trials |
| Perlis (2018)(18) | Lacked health and/or cost outcomes required for ICER calculation | Depression and anxiety |
| Ray (2015)(19) | Conference abstract | Neuropsychopharmacology |
| Roe (2018)(20) | Conference abstract | CNS Spectrums |
| RomeroPrada (2017)(21) | Conference abstract | Value in Health |
| Sicras-Mainar (2014)(22) | PGx-guided cohort absent | PloS one |
| Tanner (2019)(23) | No distinct standard care cohort (congruent vs incongruent prescribing) | ClinicoEconomics and outcomes research |
| Winner (2015)(24) | Lacked health and/or cost outcomes required for ICER calculation | Current medical research and opinion |
| Yep 2014)(25) | Conference abstract | Value in Health |

*ICER* Incremental Cost-Effectiveness Ratio, *PGx Pharmacogenomics*

## Supplementary Table 7: QHES results

| **Question** | **1** | **2** | **3** | **4** | **5** | **6** | **7** | **8** | **9** | **10** | **11** | **12** | **13** | **14** | **15** | **16** | **Score** |
| --- | --- | --- | --- | --- | --- | --- | --- | --- | --- | --- | --- | --- | --- | --- | --- | --- | --- |
| Abushanab (2024)(26) | Y | Y | Y | NA | Y | Y | Y | Y | Y | Y | Y | Y | Y | Y | Y | N | 97 |
| Berm (2016)(27) | Y | Y | N | NA | Y | Y | Y | N | Y | N | Y | Y | Y | Y | Y | Y | 79 |
| Carta (2022)(28) | Y | N | Y | NA | Y | Y | Y | N | Y | N | Y | Y | Y | Y | Y | Y | 83 |
| Ghanbarian (2023)(29) | Y | N | Y | NA | Y | Y | Y | Y | Y | Y | Y | Y | Y | Y | Y | Y | 96 |
| Girardin (2019)(30) | Y | N | N | NA | Y | Y | Y | N | Y | N | Y | Y | Y | Y | Y | Y | 75 |
| Groessl (2018)(31) | Y | N | Y | Y | Y | N | Y | N | Y | N | Y | Y | Y | Y | Y | Y | 77 |
| Hornberger (2015)(32) | Y | N | Y | NA | Y | Y | Y | Y | Y | Y | Y | Y | Y | Y | Y | Y | 96 |
| Lopez-Saavedra (2024)(33) | Y | Y | Y | NA | Y | Y | Y | N | Y | N | Y | Y | Y | Y | Y | Y | 87 |
| Najafzadeh (2017)(34) | Y | N | Y | NA | Y | Y | Y | Y | Y | Y | Y | Y | Y | Y | Y | Y | 96 |
| Ninomiya (2021)(35) | Y | N | N | NA | Y | Y | Y | Y | Y | Y | Y | Y | Y | Y | Y | Y | 88 |
| Ninomiya (2022)(36) | Y | N | N | NA | Y | Y | Y | Y | Y | Y | Y | Y | Y | N | Y | Y | 82 |
| Rejon-Parrilla (2014)(37) | Y | N | Y | NA | Y | Y | Y | N | Y | N | Y | Y | Y | N | Y | Y | 77 |
| Skokou (2024)(38) | Y | N | Y | Y | Y | Y | Y | N | Y | N | Y | Y | Y | Y | Y | Y | 83 |
| Sluiter (2018)(39) | Y | N | Y | NA | Y | Y | Y | N | Y | N | Y | Y | Y | Y | Y | N | 80 |
| Sluiter (2019)(40) | Y | N | Y | NA | Y | Y | Y | N | Y | N | Y | Y | Y | Y | Y | Y | 83 |
| Tanner (2020)(41) | Y | N | Y | NA | Y | Y | Y | Y | Y | N | Y | Y | Y | Y | Y | Y | 90 |
| ter Hark (2025)(42) | Y | N | Y | NA | Y | Y | Y | N | Y | N | Y | Y | Y | N | Y | Y | 83 |
| Weightings | 7 | 4 | 8 | 1 | 9 | 6 | 5 | 7 | 8 | 6 | 7 | 8 | 7 | 6 | 8 | 3 |  |

*QHES* Quality of Health Economic Studies, *Y* Yes, *N* No, *NA* Not applicable

This table displays the results of the quality assessment using the Quality of Health Economic Studies (QHES) tool. The QHES checklist consists of 16 items focusing on methodological aspects of economic studies such as the study characteristics, design and findings. Each item is assigned a weight according to its significance, providing a more detailed assessment of study quality. Completion of the checklist produces a score within the range of 0 to 100, where a score of ≥75 is considered of high quality

## Supplementary Table 8: Data extraction of article information

| **Study** | **PMID/DOI** | **Journal** | **Contact Author Email** |
| --- | --- | --- | --- |
| Abushanab (2024)(26) | 39469318 | Journal of Pharmaceutical Policy and Practice | [daoud.a@qu.edu.qa](mailto:daoud.a@qu.edu.qa) |
| Berm (2016)(27) | 28033366 | PLOS One | [judith@ascacademics.com](mailto:judith@ascacademics.com) |
| Carta (2022)(28) | 35930170 | Clinical Drug Investigation | [conversa@unica.it](mailto:conversa@unica.it) |
| Ghanbarian (2023)(29) | 37963621 | Canadian Medical Association Journal | [shahzad.ghanbarian@ubc.ca](mailto:shahzad.ghanbarian@ubc.ca) |
| Girardin (2019)(30) | 29298994 | The Pharmacogenomics Journal | [francois.girardin@hcuge.ch](mailto:francois.girardin@hcuge.ch) |
| Groessl (2018)(31) | 30058980 | Journal of Managed Care & Specialty Pharmacy | [AMaciel@altheadx.com](mailto:AMaciel@altheadx.com) |
| Hornberger (2015)(32) | 26247576 | The American Journal of Managed Care | [ujch@stanford.edu](mailto:ujch@stanford.edu) |
| Lopez-Saavedra (2024)(33) | 39187185 | Journal of Affective Disorders | [francisco.abad@uam.es](mailto:francisco.abad@uam.es) |
| Najafzadeh (2017)(34) | 29110140 | PharmacoEconomics | [AMaciel@altheadx.com](mailto:AMaciel@altheadx.com) |
| Ninomiya (2021)(35) | 34230449 | Translational Psychiatry | [ikeda-ma@fujita-hu.ac.jp](mailto:ikeda-ma@fujita-hu.ac.jp) |
| Ninomiya (2022)(36) | 36313369 | Frontiers in Pharmacology | [saitou@fujita-hu.ac.jp](mailto:saitou@fujita-hu.ac.jp) |
| Rejon-Parrilla (2014)(37) | <https://doi.org/10.1016/j.hlpt.2014.08.004> | Health Policy and Technology | [jcrejon@ohe.org](mailto:jcrejon@ohe.org) |
| Skokou (2024)(38) | 38364700 | EBioMedicine | [gpatrinos@upatras.gr](mailto:gpatrinos@upatras.gr) |
| Sluiter (2018)(39) | 30384381 | European Addiction Research | [rene.sluiter@radboudumc.nl](mailto:rene.sluiter@radboudumc.nl) |
| Sluiter (2019)(40) | 30647446 | The Pharmacogenomics Journal | [rene.sluiter@radboudumc.nl](mailto:rene.sluiter@radboudumc.nl) |
| Tanner (2020)(41) | 32301648 | Pharmacogenomics | [Bryan.Dechairo@myriad.com](mailto:Bryan.Dechairo@myriad.com) |
| ter Hark (2025)(42) | 40769295 | Value in Health | [sophie.terhark@radboudumc.nl](mailto:sophie.terhark@radboudumc.nl) |

*PMID* PubMed Identifier, *DOI* Digital Object Identifier

## Supplementary Table 9: Data extraction of study characteristics

| **Study** | **Country** | **Condition** | **Perspective** | **Economic Evaluation** | **Study Design** | **Funding** | **Conflicts of Interest** |
| --- | --- | --- | --- | --- | --- | --- | --- |
| Abushanab (2024)(26) | Qatar | MDD | Healthcare | CUA/CEA^a^ | Model-based | No statement | No conflicts of interest |
| Berm (2016)(27) | The Netherlands | DEP | Healthcare insurance payer | CUA | Model-based | Non-private | Yes |
| Carta (2022)(28) | Italy | MDD | Societal | CUA | Model-based | Non-private | No conflicts of interest |
| Ghanbarian (2023)(29) | Canada | MDD | Public Payer | CUA | Model-based | Non-private | Yes |
| Girardin (2019)(30) | USA | SCZ | Third-party payer | CUA | Model-based | Non-private | No conflicts of interest |
| Groessl (2018)(31) | USA | MDD | Societal | CUA | Model-based | Private | Yes |
| Hornberger (2015)(32) | USA | MDD | Societal | CUA | Model-based | Private | Yes |
| Lopez-Saavedra (2024)(33) | Spain | MDD | Societal and healthcare provider | CUA | Model-based | Non-private | No conflicts of interest |
| Najafzadeh (2017)(34) | USA | DEP and/or ANX | Societal | CUA | Model-based | No funding | Yes |
| Ninomiya (2021)(35) | UK and Japan | SCZ | Healthcare provider | CUA | Model-based | Non-private | Yes |
| Ninomiya (2022)(36) | UK | SCZ | Third-party payer | CUA | Model-based | Non-private | Yes |
| Rejon-Parrilla (2014)(37) | UK | SCZ | Healthcare system | CUA | Model-based | No funding | No conflicts of interest |
| Skokou (2024)(38) | Greece | MDD^b^ | Healthcare system | CUA | Alongside RCT | Non-private | No conflicts of interest |
| Sluiter (2018)(39) | The Netherlands | AUD | Societal | CUA | Model-based | No statement | No conflicts of interest |
| Sluiter (2019)(40) | The Netherlands | MDD | Societal | CUA | Model-based | Non-private | No conflicts of interest |
| Tanner (2020)(41) | Canada | DEP | Healthcare system | CUA | Model-based | Private and non-private | Yes |
| ter Hark (2025)(42) | The Netherlands | MDD | Societal and healthcare | CUA | Alongside RCT | Non-private | Yes |

*USA* United States of America, *UK* United Kingdom, *DEP* Depression, *ANX* Anxiety, *MDD* Major Depressive Disorder, *SCZ* Schizophrenia, *AUD* Alcohol Use Disorder, *CUA* Cost-Utility Analysis, *CEA* Cost-Effectiveness Analysis, *RCT* Randomised Controlled Trial

This table includes study characteristics for each study included in the systematic review. These characteristics include first author, year of publication, country of target population, study perspective, type of economic evaluation, study design, whether the study received funding or not, and if they had any conflicts of interest.

^a^ Even though most studies stated they were carrying out a cost-effectiveness analysis, if their main outcome was an ICER using QALYs gained, they were categorised as a cost-utility analysis. Abushanab and colleagues carried out both a short-term model and a long-term model with different outcomes.(26) For the short-term model, the ICER was based on response without side-effects and without relapse whereas the long-term model reported ICER/years of life saved and ICER/QALYs gained, hence the CUA/CEA category.

^b^ Skokou and colleagues looked at multiple psychiatric conditions however, the cohorts of schizophrenia and bipolar disorder were excluded from their conclusions due to inaccurate self-reported quality of life and so are not included here.

## Supplementary Table 10: Data extraction of study methods

| **Study** | **Time horizon** | **Model Type** | **Time of Testing** | **PGx Testing Approach** | **Discount** | **Cost Year** | **Currency** | **Data source of input parameters** |
| --- | --- | --- | --- | --- | --- | --- | --- | --- |
| Abushanab (2024)(26) | 6 weeks/lifetime^a^ | Decision tree/Markov | Pre-emptive | Multi-gene | 3% | 2024/25 | USD/QAR | RCT and literature |
| Berm (2016)(27) | 12 weeks | Decision tree | Pre-emptive | Single-gene | NA | 2014 | EUR | Literature |
| Carta (2022)(28) | 18 weeks | Markov | Pre-emptive | Single-gene | NA | 2021 | EUR | Meta-analysis and literature |
| Ghanbarian (2023)(29) | 20 years | Discrete-time microsimulation | Reactive/pre-emptive^b^ | Multi-gene | 1.50% | 2020 | CAD | Systematic review, literature and clinical expert panel judgements |
| Girardin (2019)(30) | 3 years | Decision tree/Markov | Pre-emptive | Multi-gene | 3% | 2017 | USD | Literature |
| Groessl (2018)(31) | 3 years | Markov | Pre-emptive | Multi-gene | 3% | 2016 | USD | RCT and literature |
| Hornberger (2015)(32) | Lifetime | Markov | Pre-emptive | Multi-gene | 3% | 2013 | USD | Meta-analysis and literature |
| Lopez-Saavedra (2024)(33) | 3 years | Markov microsimulation | Pre-emptive | Multi-gene | 3% | 2019 | EUR | Meta-analysis and literature |
| Najafzadeh (2017)(34) | 3 years | Discrete-event simulation | Pre-emptive | Multi-gene | 3% | 2017 | USD | RCT and literature |
| Ninomiya (2021)(35) | 10 years | Decision tree/Markov | Pre-emptive | Multi-gene/single-gene ^c^ | 2% for Japan, 3.5% for UK | 2020 | GBP | Literature |
| Ninomiya (2022)(36) | 10 years | Decision tree/Markov | Pre-emptive | Multi-gene | 3.5% | 2019 | GBP | Literature |
| Rejon-Parrilla (2014)(37) | 2 years | Decision tree/Markov | Pre-emptive | Single-gene | 3.5% | Not specified | GBP | Literature |
| Skokou (2024)(38) | 12 weeks-19 months | Not model-based | Pre-emptive | Single-gene^d^ | NA | Not specified | EUR | RCT and literature |
| Sluiter (2018)(39) | 1 year | Markov | Pre-emptive | Single-gene | NA | 2015 | EUR | Literature |
| Sluiter (2019)(40) | 12 weeks | Markov | Pre-emptive | Single-gene | NA | 2015 | EUR | RCT and literature |
| Tanner (2020)(41) | 5 years | Decision tree/Markov | Pre-emptive | Multi-gene | 3% | 2018 | CAD | RCT and literature |
| ter Hark (2025)(42) | 13 weeks and 26 weeks | Not model-based | Pre-emptive | Multi-gene | NA | 2022 | EUR | RCT and literature |

*NA* Not applicable, *USD* United States Dollars, *QAR* Qatari Riyal, *EUR* Euro, *GBP* Great British Pounds, *CAD* Canadian Dollars, *RCT* Randomised Controlled Trial.

This table displays the study methods for each study included in the systematic review. It includes details about the first author, year of publication, time horizon used, type of model used, if a discounting factor was used and if so by how much, year of costs used, currency used and the data source for the input parameters for the model.

^a^ Abushanab and colleagues developed two models, one short-term and one long-term.(26)

^b^ For the study by Ghanbarian and colleagues, patients with prevalent MDD received pharmacogenomics (PGx) testing before any prescription but patients with a new diagnosis of MDD would receive PGx testing after one unsuccessful medication trial so was categorised as both pre-emptive and reactive.(29)

^c^ The study by Ninomiya and colleagues simulated a Japanese cohort and a cohort from the UK.^31^ For the Japanese cohort only the HLA-B*59:01 variant was considered whereas for the cohort from the UK, both HLA-B 158T and HLA-DQB1 126Q were evaluated

^d^ The economic evaluation by Skokou and colleagues was a part of the PREPARE trial which evaluated a pre-emptive multi-gene panel however the PGx-guided arm of this economic evaluation received treatment based only on CYP2D6 or CYP2C19 genotyping results so was classed as single-gene testing.^35^

## Supplementary Table 11: Data extraction of study uncertainty assessments, willingness-to-pay thresholds, and outcomes

| **Study** | **DSA** | **PSA** | **Scenario Analysis** | **Subgroup Analysis** | **WTP Threshold** | **Cost** | **LY** | **QALY** | **ICER^a^** |
| --- | --- | --- | --- | --- | --- | --- | --- | --- | --- |
| Abushanab (2024)(26) | X | X | X |  | 150 000 USD (546 000 QAR) | X | X | X | X |
| Berm (2016)(27) | X | X | X |  | 50 000 EUR | X |  | X | X |
| Carta (2022)(28) | X | X | X |  | 75 000 EUR | X |  | X | X |
| Ghanbarian (2023)(29) | X | X |  |  | 50 000 CAD | X | X | X | X |
| Girardin (2019)(30) | X | X |  |  | Not clear | X |  | X | X |
| Groessl (2018)(31) | X |  |  | X | 50 000 USD | X |  | X |  |
| Hornberger (2015)(32) | X | X |  |  | 50 000 USD | X |  | X | X |
| Lopez-Saavedra (2024)(33) | X | X | X |  | 50 000 EUR | X |  | X | X |
| Najafzadeh (2017)(34) | X | X |  |  | 50 000 USD | X |  | X | X |
| Ninomiya (2021)(35) | X | X | X |  | 37 650.6 GBP (5 000 000 JPY) and 30 000 GBP | X |  | X | X |
| Ninomiya (2022)(36) | X | X | X |  | 30 000 GBP | X |  | X | X |
| Rejon-Parrilla (2014)(37) | X |  | X |  | Refers to NICE cost-effectiveness requirements of £20 000-30 000 | X |  | X | X |
| Skokou (2024)(38) |  | X |  | X | Not clear | X |  | X | X |
| Sluiter (2018)(39) | X | X |  |  | 80 000 EUR | X |  | X | X |
| Sluiter (2019)(40) | X | X | X |  | 80 000 EUR | X |  | X | X |
| Tanner (2020)(41) | X | X | X |  | 50 000 CAD | X |  | X | X |
| ter Hark (2025)(42) | X |  | X |  | 50 000 EUR | X |  | X | X |

*DSA* Deterministic Sensitivity Analysis, *PSA* Probabilistic Sensitivity Analysis, *WTP* Willingness-to-pay, *USD* United States Dollars, *QAR* Qatari Riyal, *EUR* Euro, *GBP* Great British Pounds, *CAD* Canadian Dollars, *JPY* Japanese Yen, *LY* Life-Year, *QALY* Quality-Adjusted Life Year, *ICER* Incremental Cost-Effectiveness ratio

This study includes information about the types of uncertainty analyses completed, the willingness-to-pay threshold used and the outcomes for each of the studies included in the systematic review.

^a^ Marked as ‘X’ if the study carried out an Incremental Cost-Effectiveness Ratio, even if they did not provide the value due to it being negative.

## Supplementary Table 12: Data extraction of patients and intervention information

| **Study** | **Target population** | **Cohort** | **Sample Size** | **Age** | **% Male** | **Drug(s)** |
| --- | --- | --- | --- | --- | --- | --- |
| Abushanab (2024)(26) | Treatment-naïve patients with MDD who scored 20 or higher on the HAM-D17 | Hypothetical | 15 000 | Mean age: 48 years old | Not specified | Paroxetine, Vortioxetine, Fluvoxamine, Venlafaxine, Escitalopram, Sertraline |
| Berm (2016)(27) | Elderly depressive patients treated with nortriptyline | Hypothetical | 2 000 (1 000 PGx, 1 000 SoC) | ≥60 years old | Not specified | Nortriptyline, Tranylcypromine |
| Carta (2022)(28) | Patients diagnosed with MDD | Hypothetical | 2 000 (1 000 PGx, 1 000 SoC) | Adult | Not specified | Not specified |
| Ghanbarian (2023)(29) | Patients with newly diagnosed and prevalent MDD | Hypothetical | 194 149 | Mean age: 45.6 years old | Prevalent patients: 32%, newly diagnosed patients: 42% | 40 different antidepressants indicated for MDD treatment in the CANMAT guideline |
| Girardin (2019)(30) | Adults with TRS who received clozapine as a third-line anti-psychotic medication | Hypothetical | 10 000 | Adult | Not specified | Clozapine |
| Groessl (2018)(31) | Treatment-naive patients with MDD or patients with inadequately controlled MDD and a score of 20 or greater on the HAM-D17. | Hypothetical | Not specified | 48 years old | Not specified | Not specified |
| Hornberger (2015)(32) | MDD patients nonresponsive to 1 or more treatment | Hypothetical | Not specified | Mean age: 44 years old | Not specified | Not specified |
| Lopez-Saavedra (2024)(33) | MDD patients who are naïve to treatment | Hypothetical | Not specified | Not specified | Not specified | Not specified |
| Najafzadeh (2017)(34) | Patients with moderate or severe depression (HAM-D ≥ 20) and/or anxiety (HAM-A ≥ 18) | Hypothetical | 2 000 (1 000 PGx, 1 000 SoC) | Mean age: 48 years old | 27% | Not specified |
| Ninomiya (2021)(35) | Adults from Japan and the UK with TRS who are eligible for clozapine treatment | Hypothetical | Not specified | Mean age: 40.6 years old | Not specified | Clozapine |
| Ninomiya (2022)(36) | TRS patients who are eligible for clozapine treatment | Hypothetical | Not specified | Not specified | Not specified | Clozapine |
| Rejon-Parrilla (2014)(37) | First episode schizophrenia patients being prescribed risperidone | Hypothetical | Not specified | Mean age: 25 years old | Not specified | Risperidone |
| Skokou (2024)(38) | Adults with a clinical diagnosis of a psychiatric disorder, namely psychosis, MDD and bipolar disorder that were treatment naïve to 13 psychiatric medications with proven clinical actionability based on DPWG guidelines | RCT | 1 076 (547 PGx, 529 SoC)  SCZ: 330 (147 PGx, 183 SoC) MDD: 494 (261 PGx, 233 SoC) Bipolar: 252 (139 PGx, 113 SoC) | Mean age PGx: 48 years old  Mean age SoC: 47 years old | PGx: 47.90%, SoC: 50.47% | Amitriptyline, Aripiprazole, Carbamazepine, Citalopram, Clomipramine, Doxepine, Escitalopram, Haloperidol, Paroxetine, Pimozide, Sertraline, Venlafaxine, Zuclopenthixol |
| Sluiter (2018)(39) | Patients with alcohol use disorders | Hypothetical | Not specified | Not specified | Not specified | Naltrexone or Acamprosate |
| Sluiter (2019)(40) | Patients with MDD in primary care before the start of antidepressant drug treatment | Hypothetical | 1 000 patients | ≥ 18 years old | Not specified | Fluoxetine, citalopram, venlafaxine, mirtazapine, amitriptyline, nortriptyline |
| Tanner (2020)(41) | Patients with moderate-to-severe depression undergoing antidepressant treatment | Hypothetical | Not specified | Starting age of 32 years old | Not specified | Not specified |
| ter Hark (2025)(42) | Patients are in- and outpatients with a primary diagnosis of severe MDD and a Hamilton Rating Scale for Depression score ≥ 19, aged 18-65 years, who are eligible for treatment with a TCA | RCT | 111 patients | 18-65 years old | PGx: 50.45%, SoC: 49.55% | Nortriptyline, Imipramine and Clomipramine |

*MDD* Major Depressive Disorder, *TRS* Treatment-Resistant Schizophrenia, *HAM-D17* 17-item Hamilton Depression Rating Scale, *HAM-A* Hamilton Anxiety Rating Scale, *DPWG* Dutch Pharmacogenetics Working Group, *PGx* Pharmacogenomics, *SoC* Standard of care, *CANMAT* Canadian Network for Mood and Anxiety Treatments, *RCT* Randomised Controlled Trial, *TCA* Tricyclic Antidepressant

This table includes information about the patients and interventions for each of the studies in the systematic review. This includes the first author, year of publication, target population, type of patient cohort, sample size, age of patient cohort, percentage of males and drug(s) evaluated in the study.

## Supplementary Table 13: Summary estimates extracted from full-text papers

| **Study** | **Sub-groups** | **PGx Cost (95% CI/SD)** | **SoC Cost (95% CI/SD)** | **∆C (95% CI/SD)** | **PGx QALY (95% CI/SD)** | **SoC QALY (95% CI/SD)** | **∆E (95% CI/SD)** | **ICER (95% CI/SD)** |
| --- | --- | --- | --- | --- | --- | --- | --- | --- |
| Abushanab (2024)(26) | Lifetime model | 116 922 | 163 137 | -46 215  (-15 744-101 758) | 927 | 866 | 61 (-285-457) (0.06 per person) | -757.62 |
| Berm (2016)(27) | - | 7 528 292 | 7 374 826 | 153 466 | 4.46 | 4.57 | 0.12 | 1 333 148 |
| Carta (2022)(28) | CYP2D6 | 802.23 | 698.24 | 104 | 0.2192 | 0.217 | 0.0022 | 46 908.24 |
|  | CYP2C19 | 776.07 | 655.84 | 120.22 | 0.221 | 0.219 | 0.0020 | 60 094.17 |
| Ghanbarian (2023)(29) | - | 8 960 000 000 | 9 920 000 000 | 956 000 000  (-4 926 per patient) | 2 234 977 | 2 160 954 | 74 023 (0.381 per person) | -12 929.13 |
| Girardin (2019)(30) | - | 13 091  (7 154 to 19 023) | 13 694 (7 752-19 626) | -603 | 1.8350^c^ (1.69-1.97) | 1.8353 (1.69-1.97) | -0.0003 | 3 930 000  (2 010 000-8 170 000) |
| Groessl (2018)(31) | Moderately-severely depressed | 44 697 | 47 295 | -2 598 | 2.07 | 1.97 | 0.10 | -25 980 |
|  | Severely depressed | 41 215 | 47 025 | -5 810 | 2.15 | 1.98 | 0.17 | -34 176.47 |
| Hornberger (2015)(32) | - | 272 751 | 276 515 | -3 764 | 13.624 | 13.308 | 0.316 | -11 911.39 |
| Lopez-Saavedra (2024)(33) | Societal | 1 687.02 (84.05) | 3 172.85 (151.22) | -1 485.83 | 2.84 (0.01) | 2.64 (0.02) | 0.2 | -7 820.56 |
|  | Healthcare provider | 446.60 (21.85) | 662.62 (42.07) | -216.02 | 2.84 (0.02) | 2.64 (0.02) | 0.2 | -1 130.16 |
| Najafzadeh (2017)(34) | - | 14 124  (10 703-17 630) | 14 659 (10 384-19 275) | -535  (-2 902-1 692) | 2.09 (1.88-2.28) | 1.94 (1.66 to 2.21) | 0.15 (0.04-0.28) | -3 566.67 |
| Ninomiya (2021)(35) | Japan | 16 552 | 16 487 | 65 | 6.22917 | 6.22608 | 0.00309 | 21 024 |
|  | UK | 4 281 | 4 211 | 70 | 5.8299 | 5.82665 | 0.00325 | 21 343 |
| Ninomiya (2022)(36) | - | - | - | - | - | - | - | 16 215 |
| Rejon-Parrilla (2014)(37) | - | 10 656 | 8 583 | 2 059 | 1.23 | 1.12 | 0.11 | 19 252 |
| Skokou (2024)(38) | Schizophrenia | 1 243 | 1 115 | 128 | 0.9697 | 0.9768 | -0.0071 | -18 028 |
|  | MDD | 1 302 | 2 526 | -1 224 | 0.935 | 0.925 | 0.01 | -122 400 |
|  | Bipolar disorder | 940 | 1 027 | -87 | 0.96 | 0.97 | -0.01 | 8 700 |
| Sluiter (2018)(39) | - | 3 610.66  (1 666-5 681) | 3 544.44 (1 024-5 629) | 66.22 (-28-49) | 0.714 (0.574-0.862) | 0.709 (0.567-0.862) | 0.005 (0.000-0.011) | 13 349.71 (442 000 to dominant) |
| Sluiter (2019)(40) | - | 970 (799 to 1 244) | 881 (723 to 1 130) | 89 (39-152) | 0.146 (0.133-0.159) | 0.145 (0.132-0.157) | 0.001 (0.001-0.002) | 77 406 (22 500-277 500) |
| Tanner (2020)(41) | - | - | - | -2 431 | - | - | 0.168 | -14 454 |

| ter Hark (2025)(42) | 13 weeks healthcare | 9 204  (4 000 – 15 769) | 9 352  (3 505 – 15 836) | -148  (-3 133 – 2 594) | 0.115  (0.036 – 0.176) | 0.127  (0.049 – 0.189) | -0.0125  (-0.040 – 0.0149) | 11 840 |
| --- | --- | --- | --- | --- | --- | --- | --- | --- |
| ter Hark (2025)(42) | 13 weeks societal | 18 394  (9 297 – 29 937) | 17 094  (7 069 – 29 751) | 1 300  (-3 121 – 5 191) | 0.115  (0.036 – 0.176) | 0.127  (0.049 – 0.189) | -0.0125  (-0.040 – 0.0149) | -104 000 |
| ter Hark (2025)(42) | 26 weeks healthcare | 14 018  (6 281 – 24 484) | 14 539  (5 987 – 25 997) | -521  (-5 051 – 3 539) | 0.248  (-0.093 – 0.377) | 0.247  (0.086 – 0.385) | 0.0012  (-0.049 – 0.0574) | -434 166.67 |
| ter Hark (2025)(42) | 26 weeks societal | 31 328  (16 156 – 51 050) | 29 624  (12 636 – 50 442) | 1 704  (-5 481 – 8 045) | 0.248  (-0.093 – 0.377) | 0.247  (0.086 – 0.385) | 0.0012  (-0.049 – 0.0574) | 1 420 000 |

*PGx* Pharmacogenomics, *SoC* Standard of Care, *CI* Confidence Intervals, *SD* Standard deviation, *∆E* Incremental effect, *∆C* Incremental cost, *QALY* Quality-Adjusted Life Year, *ICER* Incremental Cost-Effectiveness Ratio, *MDD* Major Depressive Disorder.

Data extracted from full-text versions of the paper including economic outcomes, health outcomes, and the Incremental Cost-Effectiveness Ratio (ICER). Uncertainty measures such as 95% confidence intervals or standard error are included when provided.

^a^ Gray shaded cells were values calculated using data provided in the full-text paper as they were not provided.

^b^ Diagonal line shading in cells indicate studies that provided outcomes per population rather than per person.

^c^ The study by Girardin and colleagues used quality-adjusted life-days (QALDs) as their outcome measure rather than quality-adjusted life-years (QALYs). To be able to compare these results to the rest of the economic evaluations, we converted the QALDs to QALYs by dividing by 365.(30)

## Supplementary Table 14: Data used to calculate incremental net benefit and its variance

| **Study** | **∆E mean** | **∆C mean** | **Var(∆E)** | **Var(∆C)** | **Covariance** | **Currency** | **Year of currency** | **∆C mean 2024 (GBP)** | **WTP** | **WTP 2024 (GBP)** | **INB** | **Var(INB)** |
| --- | --- | --- | --- | --- | --- | --- | --- | --- | --- | --- | --- | --- |
| Carta (2022a)(28) | 0.00 | 103.42 | 0.00 | 3 091.66 | -0.10 | EUR | 2021 | 113.62 | 75 000 | 82 398.02 | 75.30 | 56 246.30 |
| Carta (2022b)(28) | 0.00 | 121.61 | 0.00 | 1 825.91 | -0.04 | EUR | 2021 | 133.61 | 75 000 | 82 398.02 | 29.52 | 21 316.80 |
| Hornberger (2015)(32) | 0.33 | -5 124.23 | 0.06 | 28 176 429.45 | -224.25 | USD | 2013 | -4 714.43 | 50 000 | 46 001.35 | 19 804.16 | 174 167 903.94 |
| Lopez-Saavedra (2024a)(33) | 0.19 | -1 485.54 | 0.00 | 27 736.13 | -1.28 | EUR | 2019 | -1 909.65 | 21 000 | 26 995.19 | 7 076.35 | 400 435.68 |
| Lopez-Saavedra (2024b)(33) | 0.19 | -215.52 | 0.00 | 2 100.46 | -0.33 | EUR | 2019 | -277.04 | 21 000 | 26 995.19 | 5 457.31 | 337 551.66 |
| Ninomiya (2021a)(35) | 0.00 | 64.00 | 0.00 | 1 110.91 | -0.01 | GBP | 2020 | 73.89 | 37 651 | 34 637.08 | 36.10 | 3 979.08 |
| Ninomiya (2021b)(35) | 0.00 | 67.34 | 0.00 | 72.40 | -0.01 | GBP | 2020 | 83.59 | 30 000 | 34 637.08 | 39.54 | 3 128.99 |
| Ninomiya (2022)(36) | 0.00 | 69.01 | 0.00 | 105.42 | -0.01 | GBP | 2019 | 84.41 | 30 000 | 36 692.10 | 76.20 | 4 896.73 |
| Sluiter (2018)(39) | 0.01 | 35.45 | 0.00 | 3 785.31 | -0.20 | EUR | 2015 | 40.29 | 80 000 | 90 930.92 | 582.27 | 170 110.93 |
| Sluiter (2019)(40) | 0.00 | 93.51 | 0.00 | 1 197.85 | 0.00 | EUR | 2015 | 106.29 | 80 000 | 90 930.92 | 4.39 | 4 384.15 |
| Tanner (2020)(41) | 0.19 | -3 296.02 | 0.02 | 6 484 457.42 | -162.22 | CAD | 2018 | -2 327.42 | 50 000 | 35 306.56 | 8 902.14 | 48 171 058.65 |

*∆E* Incremental effect, *∆C* Incremental cost, *Var(∆E)* Variance of incremental effect, *Var(∆C)* Variance of incremental cost, *EUR* Euro, *USD* United States Dollar, *GBP* Great British Pounds, *CAD* Canadian Dollar, *WTP* Willingness-to-Pay Threshold, *INB* Incremental Net Benefit, *Var(INB)* Variance of incremental net benefit

The values used to calculate the Incremental Net Benefit (INB) and its variance for the meta-analysis. The data includes the mean incremental cost (∆C) and incremental effect (∆E) of pharmacogenomics-guided prescribing for psychiatric disorders compared to standard care. These values were calculated from the distributions extracted from cost-effectiveness planes provided by the studies. Currency data and the willingness-to-pay threshold were extracted from the paper and converted to 2024 Great British Pounds to ensure comparability. In some cases, multiple study groups used in the meta-analysis were derived from a single study, Carta (2022a)(28) focuses on pharmacogenetic-testing of CYP2D6, while Carta (2022b)(28) examines CYP2C19, Lopez-Saavedra (2024a)(33) evaluates a societal perspective, whereas Lopez-Saavedra (2024b)(33) uses a healthcare provider perspective; and Ninomiya (2021a)(35) is simulating a Japanese population, and Ninomiya (2021b)(35) is simulating a UK population.

## Supplementary Table 15: Egger’s regression-based test for total study groups

Egger’s regression-based test for the meta-analysis of total 11 study groups from eight studies using a random-effects model:

| **Egger's Regression-Based Test^a^** | | | | | | |
| --- | --- | --- | --- | --- | --- | --- |
| Parameter | Coefficient | Std. Error | t | Sig. (2-tailed) | 95% Confidence Interval | |
|  |  |  |  |  | Lower | Upper |
| (Intercept) | 958.186 | 813.1096 | 1.178 | .269 | -881.195 | 2797.568 |
| SE^b^ | 1.671 | .7394 | 2.260 | .050 | -.002 | 3.344 |
| a. Random-effects meta-regression | | | | | | |
| b. Standard error of effect size | | | | | | |

## Supplementary Table 16: Egger’s regression-based test for homogeneous subgroup using fixed-effects model

Egger’s regression-based test for the meta-analysis of homogenous eight study groups from six studies using a fixed-effects model:

| **Egger's Regression-Based Test^a^** | | | | | | |
| --- | --- | --- | --- | --- | --- | --- |
| Parameter | Coefficient | Std. Error | t | Sig. (2-tailed) | 95% Confidence Interval | |
|  |  |  |  |  | Lower | Upper |
| (Intercept) | -28.434 | 54.4459 | -.522 | .620 | -161.658 | 104.791 |
| SE^b^ | .979 | .6308 | 1.552 | .172 | -.565 | 2.522 |
| a. Fixed-effects meta-regression | | | | | | |
| b. Standard error of effect size | | | | | | |

## Supplementary Table 17: Egger’s regression-based test for homogeneous subgroup using random-effects model

Eggers regression-based test for the meta-analysis of homogenous eight study groups from six studies using a random-effects model:

| **Egger's Regression-Based Test^a^** | | | | | | |
| --- | --- | --- | --- | --- | --- | --- |
| Parameter | Coefficient | Std. Error | t | Sig. (2-tailed) | 95% Confidence Interval | |
|  |  |  |  |  | Lower | Upper |
| (Intercept) | -28.434 | 54.4459 | -.522 | .620 | -161.658 | 104.791 |
| SE^b^ | .979 | .6308 | 1.552 | .172 | -.565 | 2.522 |
| a. Random-effects meta-regression | | | | | | |
| b. Standard error of effect size | | | | | | |

# Supplementary Figures

## Supplementary Figure 1: Subgroup analysis by testing approach

**
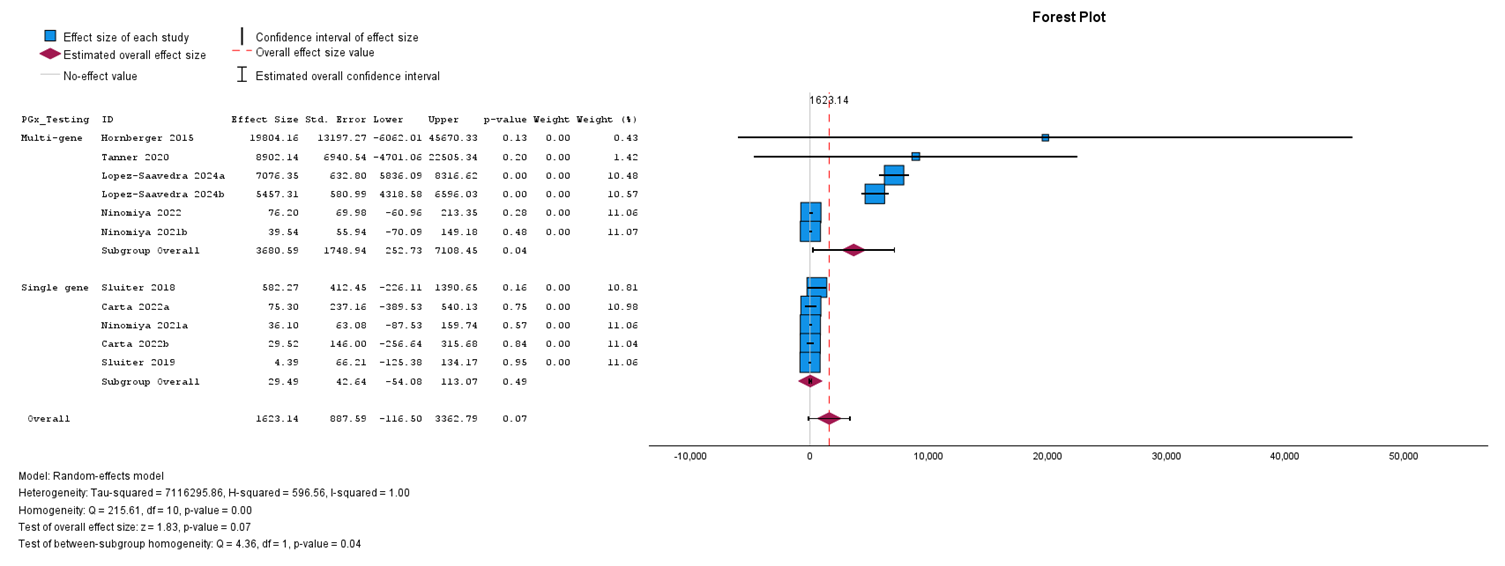
**

This forest plot shows a subgroup meta-analysis of effect sizes (Incremental Net Benefit) by the type of testing used, either multi-gene testing or single-gene testing. Each study is represented by a blue square indicating the point estimate of the effect size with a horizontal line representing the 95% confidence intervals (CIs). Subgroup and the overall pooled effect sizes are shown as red diamonds. The vertical dashed red line indicates the overall pooled effect size, also with horizontal. 95% CI’s. In some cases, multiple study groups used in the meta-analysis were derived from a single study, Carta (2022a)(28) focuses on pharmacogenetic-testing of CYP2D6, while Carta (2022b)(28) examines CYP2C19, Lopez-Saavedra (2024a)(33) evaluates a societal perspective, whereas Lopez-Saavedra (2024b)(33) uses a healthcare provider perspective; and Ninomiya (2021a)(35) is simulating a Japanese population, and Ninomiya (2021b)(35) is simulating a UK population.

## Supplementary Figure 2: Subgroup analysis by psychiatric disorder

**
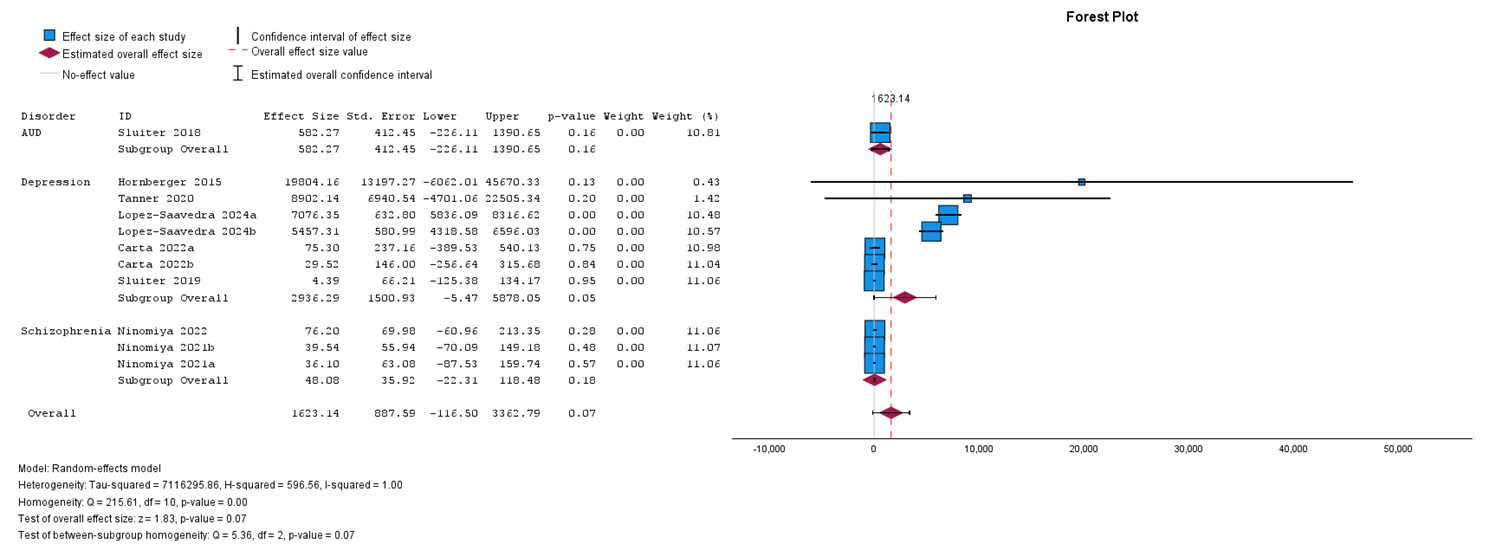
**

This forest plot shows a subgroup meta-analysis of effect sizes (Incremental Net Benefit) by psychiatric disorders, including alcohol use disorder, depression, and schizophrenia. Each study is represented by a blue square indicating the point estimate of the effect size with a horizontal line representing the 95% confidence intervals (CIs). Subgroup and the overall pooled effect sizes are shown as red diamonds. The vertical dashed red line indicates the overall pooled effect size, also with horizontal. 95% CI’s. In some cases, multiple study groups used in the meta-analysis were derived from a single study, Carta (2022a)(28) focuses on pharmacogenetic-testing of CYP2D6, while Carta (2022b)(28) examines CYP2C19, Lopez-Saavedra (2024a)(33) evaluates a societal perspective, whereas Lopez-Saavedra (2024b)(33) uses a healthcare provider perspective; and Ninomiya (2021a)(35) is simulating a Japanese population, and Ninomiya (2021b)(35) is simulating a UK population.

## Supplementary Figure 3: Subgroup analysis by time horizon

**
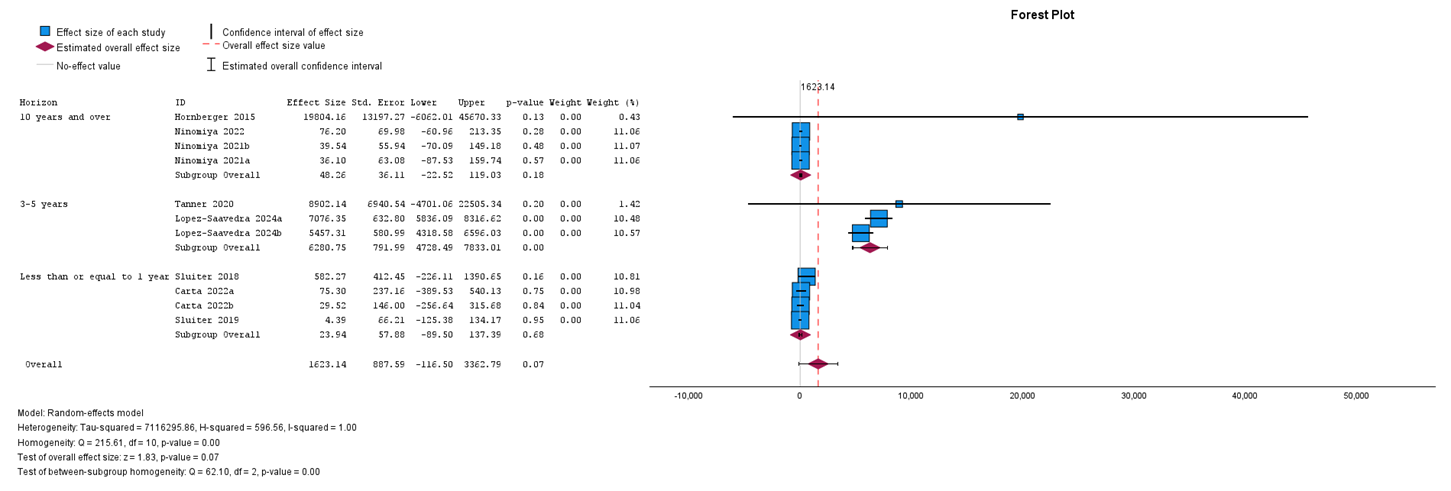
**

This forest plot shows a subgroup meta-analysis of effect sizes (Incremental Net Benefit) across various time horizons: ≤1 years; 3-5 years; and ≥10 years. Each study is represented by a blue square indicating the point estimate of the effect size with a horizontal line representing the 95% confidence intervals (CIs). Subgroup and the overall pooled effect sizes are shown as red diamonds. The vertical dashed red line indicates the overall pooled effect size, also with horizontal. 95% CI’s. In some cases, multiple study groups used in the meta-analysis were derived from a single study, Carta (2022a)(28) focuses on pharmacogenetic-testing of CYP2D6, while Carta (2022b)(28) examines CYP2C19, Lopez-Saavedra (2024a)(33) evaluates a societal perspective, whereas Lopez-Saavedra (2024b)(33) uses a healthcare provider perspective; and Ninomiya (2021a)(35) is simulating a Japanese population, and Ninomiya (2021b)(35) is simulating a UK population.

## Supplementary Figure 4: Subgroup analysis by willingness-to-pay threshold


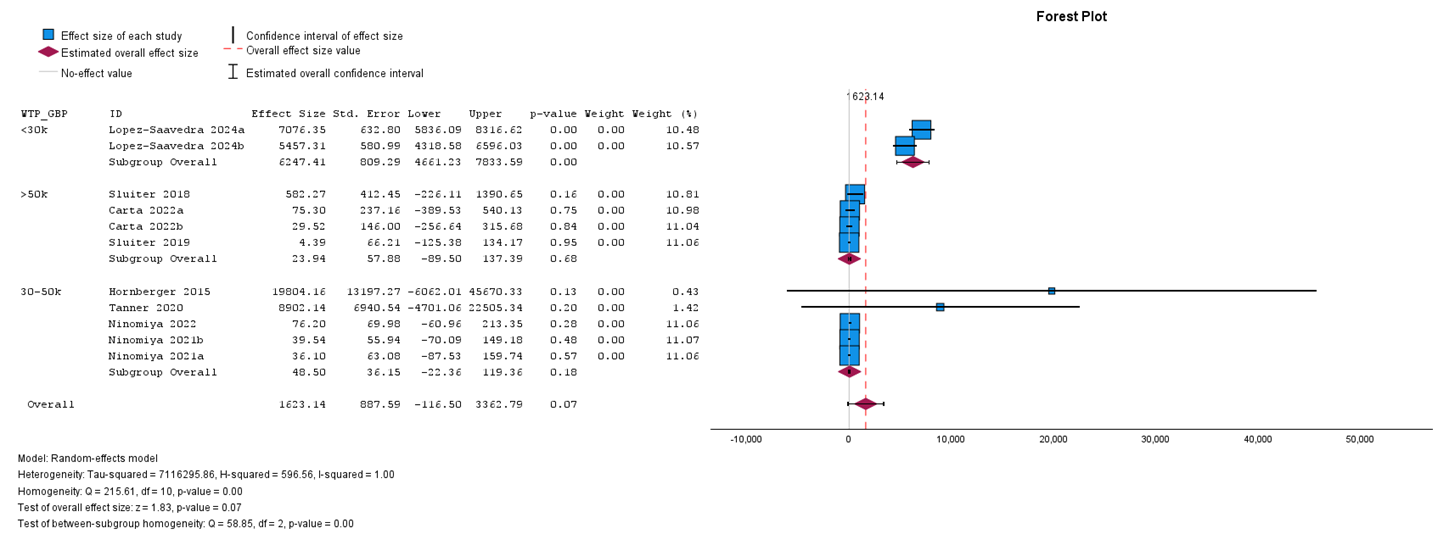


This forest plot shows a subgroup meta-analysis of effect sizes (Incremental Net Benefit) across various willingness-to-pay thresholds: <£30k, £30-50k, >50k. Each study is represented by a blue square indicating the point estimate of the effect size with a horizontal line representing the 95% confidence intervals (CIs). Subgroup and the overall pooled effect sizes are shown as red diamonds. The vertical dashed red line indicates the overall pooled effect size, also with horizontal. 95% CI’s. In some cases, multiple study groups used in the meta-analysis were derived from a single study, Carta (2022a)(28) focuses on pharmacogenetic-testing of CYP2D6, while Carta (2022b)(28) examines CYP2C19, Lopez-Saavedra (2024a)(33) evaluates a societal perspective, whereas Lopez-Saavedra (2024b)(33) uses a healthcare provider perspective; and Ninomiya (2021a)(35) is simulating a Japanese population, and Ninomiya (2021b)(35) is simulating a UK population.

## Supplementary Figure 5: Forest plot on the cost-effectiveness of pharmacogenomics-guided prescribing for psychiatric disorders for a homogenous subgroup of studies using a fixed-effects model


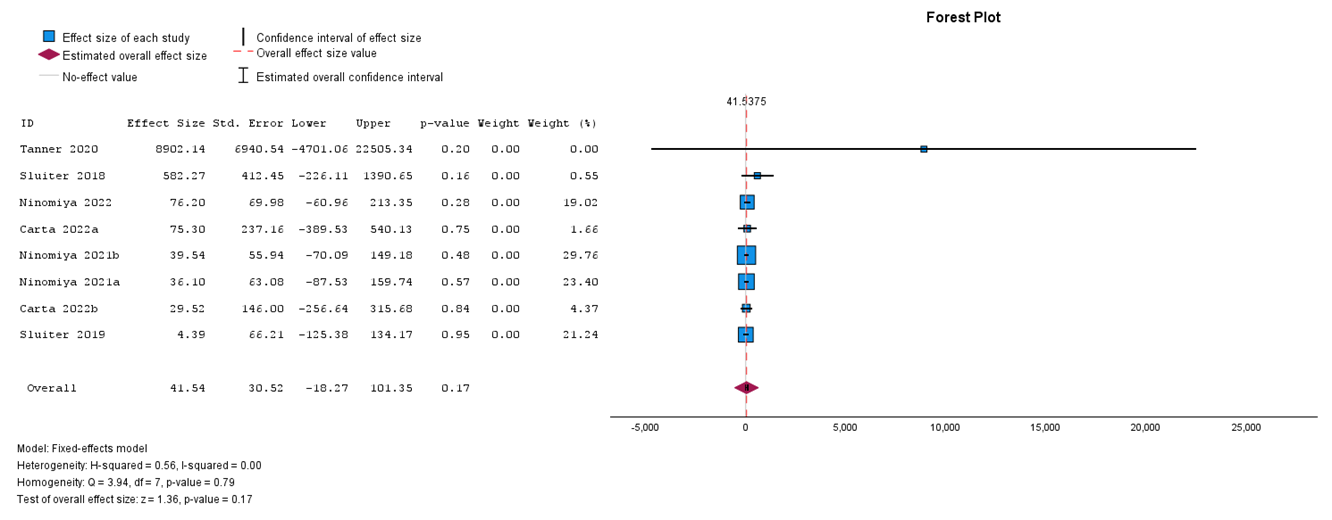


This forest plot shows the effect sizes (Incremental Net Benefit) from a meta-analysis of pharmacogenomics-guided prescribing compared to standard care for a subgroup of statistically homogenous studies using a fixed-effects model. Each study is represented by a blue square indicating the point estimate of the effect size with a horizontal line representing the 95% confidence intervals (CIs). The vertical dashed red line indicates the overall pooled effect size (£41.54), also with horizontal. 95% CIs. The analysis uses a fixed-effect model as heterogeneity was not significant. In some cases, multiple study groups used in the meta-analysis were derived from a single study: Carta (2022a)^25^ focuses on pharmacogenetic-testing of CYP2D6, while Carta (2022b)^25^ examines CYP2C19; and Ninomiya (2021a)^31^ is simulating a Japanese population, and Ninomiya (2021b)^31^ is simulating a UK population. Heterogeneity was assessed using various tests including I^2^, H^2^, and Cohran’s Q test.

## Supplementary Figure 6: Forest plot on the cost-effectiveness of pharmacogenomics-guided prescribing for psychiatric disorders for a homogenous subgroup of studies using a random-effects model

**
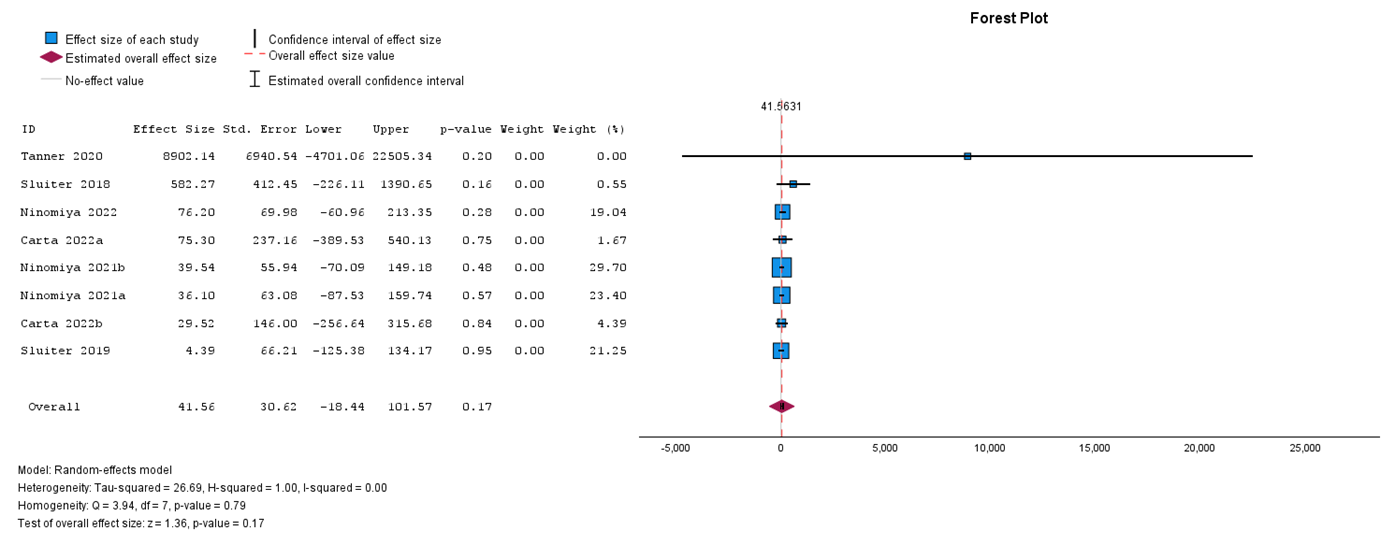
**

This forest plot shows the effect sizes (Incremental Net Benefit) from a meta-analysis of pharmacogenomics-guided prescribing compared to standard care for a subgroup of statistically homogenous studies using a random-effects model. Each study is represented by a blue square indicating the point estimate of the effect size with a horizontal line representing the 95% confidence intervals (CIs). The vertical dashed red line indicates the overall pooled effect size (£41.56), also with horizontal. 95% CIs. The analysis uses a fixed-effect model as heterogeneity was not significant. In some cases, multiple study groups used in the meta-analysis were derived from a single study: Carta (2022a)(28) focuses on pharmacogenetic-testing of CYP2D6, while Carta (2022b)(28) examines CYP2C19, Ninomiya (2021a)(35) is simulating a Japanese population, and Ninomiya (2021b)(35) is simulating a UK population.

## Supplementary Figure 7: Funnel and Galbraith plots for statistically homogenous meta-analysis

**A**

**INB**

15,000

10,000

5,000

0

-5,000

-10,000

-15,000

0

2,000

4,000

6,000

Ninomiya 2021a

Ninomiya 2021b

Carta 2022a

Ninomiya 2022

Sluiter 2018

Tanner 2020

Estimated overall effect size

(observed studies)

95% pseudo confidence

intervals

Primary studies

**Standard error**

**Inverse standard error**

0.020

0.015

0.010

0.005

0.000

3

2

1

0

-1

-2

Sluiter 2019

Carta 2022b

Ninomiya 2021a

Ninomiya 2021b

Carta 2022a

Ninomiya 2022

Sluiter 2018

Tanner 2020

95% confidence interval region

Regression line

Primary studies

**B**

**Z score**

Funnel and Galbraith plots assessing heterogeneity and publication bias for statistically homogenous subgroup of studies. (A) The funnel plot shows visual asymmetry with the study by Tanner and colleagues(41) separating from other studies (B) The Galbraith plot shows all studies falling within the 95% confidence interval region.

# Supplementary Methods

## Review protocol

| **Title of the review** | Economic Effectiveness of Pharmacogenomics-Guided Prescribing for Psychiatric Disorders: A Systematic Review and Meta-Analysis |
| --- | --- |
| **First reviewer** | Ellen Mason |
| **Team of reviewers** | Mohamed Ali |
| **Project Supervisors** | Dr Catriona Kelly  Prof David Gibson  Dr Elaine Murray  Dr Richéal Burns |

| **Support** – any advice/training for each stage | |
| --- | --- |
| **SR overview** | Atlantic Technological University (ATU) Systematic literature review and meta-analysis (iNOTE) course and Cochrane Interactive Learning: Conducting an Intervention Review |
| **Protocol development** | Cochrane Interactive Learning: Conducting an Intervention Review |
| **Literature searching** | ATU Systematic literature review and meta-analysis (iNOTE) course, Cochrane Interactive Learning: Conducting an Intervention Review and appointment with university subject librarian |
| **Quality appraisal** | ATU Systematic literature review and meta-analysis (iNOTE) course, Cochrane Interactive Learning: Conducting an Intervention Review, and Evidence Synthesis for Economic Evaluation by Evidence Synthesis Ireland. |
| **Data Extraction** |  |
| **Synthesis** |  |
| **Writing up** |  |

| **Systematic Review question** | Is Pharmacogenomics (PGx)-guided prescribing of medications for psychiatric disorders more cost-effective than standard care? |
| --- | --- |

| 1. **Specific objective(s)** |
| --- |
| To add to the current evidence base by carrying out both a narrative synthesis and meta-analysis on the cost-effectiveness of PGx-guided prescribing for adults with a diagnosed psychiatric disorder compared to standard care. This review will build upon a previous systematic review and aims to address the gap in quantitative synthesis. |

| 1. **Inclusion and exclusion criteria for studies in the review** | | |
| --- | --- | --- |
|  | **Inclusion** | **Exclusion** |
| **Population** | Adults 18 years or over with any clinically diagnosed psychiatric disorder including but not limited to psychotic disorders, mood disorders and anxiety disorders. | Under 18 years; psychiatric disorder not clinically diagnosed; studies focusing on non-psychiatric disorders |
| **Intervention/Exposure** | PGx-guided prescribing of medications for psychiatric disorders | Studies that do not evaluate PGx-guided prescribing of medications for psychiatric disorders |
| **Comparison/Control** | Standard care without PGx-guided prescribing | Studies that do not include a group receiving standard care |
| **Outcomes** | Economic outcomes (i.e. ICER’s, cost-per QALY, cost-savings) and health related quality of life outcomes (i.e. QALY’s, LY’s gained) for both the intervention and comparison cohorts. | Any qualitative studies that do not report both economic and a health-related quality of life outcomes |
| **Context** | Primary or secondary healthcare settings | Non-clinical settings |
| **Study design** | Any clinical studies that include an economic evaluation (i.e. RCT’s, cohort studies) and any full economic evaluation studies | Studies that do not include an economic evaluation; paediatric studies; case reports; opinion pieces. |

*PGx* Pharmacogenomics; *ICER* Incremental cost-effectiveness ratio; *QALY* Quality Adjusted Life Year; *LY* Life year; *RCT* Randomised controlled trial

| 1. **(a) Search methods** | |
| --- | --- |
| **Electronic databases** | All studies will be identified through searches conducted in Ovid, using the following electronic databases:  MEDLINE  EMBASE  PsycINFO |
| **Other methods used for identifying relevant research** | Reference checking |

| 1. **Methods of review** | |
| --- | --- |
| **Review manager:** Covidence | |
| **Results screening** | Titles and abstracts will be reviewed and screened independently by two reviewers, Ellen Mason and Mohamed Ali, to identify studies that meet the inclusion criteria for the review. The full text of the eligible studies will then also be independently assessed by the same two reviewers. Any discrepancies will be resolved by a consensus discussion or through discussion with a third author. |
| **Quality assessment** | The Quality of Health Economic Studies (QHES) checklist, will be used to assess the quality of the eligible studies(43). QHES was chosen given it’s use in previous systematic reviews assessing the cost-effectiveness of PGx-guided treatment, so results from this review can be related to other reviews in this field.(44–48)  The QHES checklist consists of 16 items focusing on methodological aspects of economic studies such as the study characteristics, design and findings. Each item is assigned a weight according to it’s significance, providing a more detailed assessment of study quality. Completion of the checklist produces a score within the range of 0 to 100, where a score of ≥75 is considered of high quality.    Both reviewers will assess the quality of the included studies independently and consensus will be reached through discussion if there are any discrepancies. |
| **Data extraction** | BioMed Central (BMC) Health Services Research have provided a step-by-step process to carry out data harmonisation and meta-analytic methods for the synthesis of economic evidence.(49) Given we aim to carry out a meta-analysis if it is possible, we will use their data extraction form to ensure all required information is extracted.  They instruct that the following data should be extracted:  • General characteristics of economic evaluations (EE’s)  • Characteristics of patients  • Type of interventions and comparators  • Data needed to estimate incremental net benefit (INB) and its variance |
| **Narrative synthesis** | The narrative synthesis aims to summarise and interpret the findings from the included studies regarding the cost-effectiveness of PGx-guided treatment for psychiatric disorders.  We will follow the general framework on how to conduct a narrative synthesis developed as part of the Economic and Social Research Council (ESRC) to increase transparency of our synthesis.(50) The general framework consists of four elements:   1. Theorising why and for whom the intervention will work and in this case be cost-effective for 2. Summarising the key findings across included studies 3. Exploring the relationships within and between studies 4. Assessment of the quality and robustness of the included studies and how this might influence the overall conclusions |
| **Meta-analysis** | We will subset the studies according to sample model type, population, country income, perspective and time horizon for example, in order to carry out a meta-analysis.  The BMC Health Services step-by-step meta-analytic process for economic evidence following data extraction is:(49)   - To standardise currencies by converting all monetary values to purchasing power parity (PPP) adjusted to Great British Pounds (GBP) for the latest year of analysis. The step-by-step process says to convert to US dollars however we would like to convert to GBP given we want to assess cost-effectiveness in the United Kingdom. - To adjust historical costs by converting reported costs from previous years to the current year using the consumer price index (CPI) of the respective country. - Calculate incremental net benefit (INB) using the formula:   INB=ΔE×K−ΔC, where  K is the willingness to pay (WTP),  ΔC is incremental cost, and  ΔE is incremental effectiveness.   - However since economic evaluations often report many different parameters, they have 5 scenarios of provided data and how to calculate the INB accordingly, which may require a slightly different equation.      - Estimate variance of the INB using variances of costs and effectiveness along with their covariance:   Var(INB) = K^2^σ^2^_ΔE_ + σ^2^_ΔC_ – 2Kσ_ΔEΔC_  Or  Var(INB) = K^2^σ^2^_ΔE_ + σ^2^_ICER_   - Pool INBs from multiple studies while stratifying by country income, model type, time horizon, and perspective to minimize heterogeneity. Choose a pooling model (fixed-effect or random-effect) based on the degree of heterogeneity assessed through statistical tests like the Cochrane Q test and I² statistic. - Conduct meta-regression analysis to explore potential sources of heterogeneity if significant differences are found in pooled INBs. - Assess publication bias using a funnel plot and the Egger’s test. |

| 1. **Presentation of results** | |
| --- | --- |
| **Result outputs** | - PRISMA flow diagram to visualise the screening process for transparency and repeatability - Quality assessment of included studies including assessing publication bias which will be outputted as a table. - Extraction table(s) covering a comprehensive overview of the included studies - A narrative synthesis to analyse the results within studies and between studies and whether they suggest PGx-guided prescribing is cost-effective or not compared to standard care - If possible, a meta-analysis which will be visualised as a forest plot, to further support conclusions of the narrative synthesis - Any additional further aids such as tables and charts to support findings |
| **Potential outputs from review** | - Paper - Poster presentation |

# References

1. The following abstracts were presented as posters at the 2014 NEI Psychopharmacology Congress. CNS Spectr. 2015 Feb;20(1):61–92.

2. Basu A, Dutta AK, Bagepally BS, Das S, Cherian JJ, Roy S, et al. Pharmacogenomics-assisted schizophrenia management: A hybrid type 2 effectiveness-implementation study protocol to compare the clinical utility, cost-effectiveness, and barriers. Boon-Peng H, editor. PLOS ONE. 2024 Apr 10;19(4):e0300511.

3. Benitez J, Cool CL, Scotti DJ. Use of Combinatorial Pharmacogenomic Guidance in Treating Psychiatric Disorders:financial impact on a health plan. Pers Med. 2018 Nov;15(6):481–94.

4. Brown LC, Lorenz RA, Li J, Dechairo BM. Economic Utility: Combinatorial Pharmacogenomics and Medication Cost Savings for Mental Health Care in a Primary Care Setting. Clin Ther. 2017 Mar;39(3):592-602.e1.

5. Callegari C, Isella C, Caselli I, Poloni N, Ielmini M. Pharmacogenetic Tests in Reducing Accesses to Emergency Services and Days of Hospitalization in Bipolar Disorder: A 2-Year Mirror Analysis. J Pers Med. 2019 Apr 30;9(2):22.

6. Carrascal-Laso L, Franco-Martín MÁ, Marcos-Vadillo E, Ramos-Gallego I, García-Berrocal B, Mayor-Toranzo E, et al. Economic Impact of the Application of a Precision Medicine Model (5SPM) on Psychotic Patients. Pharmacogenomics Pers Med. 2021 Aug;Volume 14:1015–25.

7. Chatziandreou E, Panagiotou N. EE77 Cost-Effectiveness Analysis of Pharmacogenetic-Guided Treatment in Drug Resistant Depression. Value Health. 2022 Dec;25(12):S68.

8. Conley VM, Daack-Hirsch S, Halbmaier K, Shaw L. Bringing Personalized Medicine to a PACT Program: A Quality Improvement Project. J Am Psychiatr Nurses Assoc. 2020 Jan;26(1):77–85.

9. Edwards AM, Perlis RH, Krause DS. 128 Factors Associated with Cost Savings Following Use of a Pharmacogenetic Assay in Individuals with Mood and Anxiety Disorders. CNS Spectr. 2020 Apr;25(2):281–2.

10. Espadaler J, Carcedo D, Pérez-Mitru A, Menchón J, Saiz-Ruiz J, Bobes J, et al. Cost-Consequence Analysis of Using Neurofarmagen In The Decision-Making Process During The Treatment of Patients With Depression In The U.S. Value Health. 2017 Oct;20(9):A713.

11. Fabbri C, Kasper S, Zohar J, Souery D, Montgomery S, Albani D, et al. Cost-effectiveness of genetic and clinical predictors for choosing combined psychotherapy and pharmacotherapy in major depression. J Affect Disord. 2021 Jan;279:722–9.

12. Fagerness J, Fonseca E, Hess GP, Scott R, Gardner KR, Koffler M, et al. Pharmacogenetic-Guided Psychiatric Intervention Associated With Increased Adherence and Cost Savings.

13. Jablonski MR, Lorenz R, Li J, Dechairo BM. Economic Outcomes Following Combinatorial Pharmacogenomic Testing for Elderly Psychiatric Patients. J Geriatr Psychiatry Neurol. 2020 Nov;33(6):324–32.

14. Maciel A, Cullors A, Lukowiak AA, Garces J. Estimating cost savings of pharmacogenetic testing for depression in real-world clinical settings. Neuropsychiatr Dis Treat. 2018 Jan;Volume 14:225–30.

15. Mayhew M, Jablonski M, Li J, Dechairo B. Combinatorial Pharmacogenomics Reduces Polypharmacy and Medication Cost in Elderly Patients with Anxiety and Depression. Am J Geriatr Psychiatry. 2017 Mar;25(3):S143–4.

16. Olson MC, Maciel A, Gariepy JF, Cullors A, Saldivar JS, Taylor D, et al. Clinical Impact of Pharmacogenetic-Guided Treatment for Patients Exhibiting Neuropsychiatric Disorders: A Randomized Controlled Trial. Prim Care Companion CNS Disord [Internet]. 2017 Mar 16 [cited 2025 July 2];19(02). Available from: https://www.psychiatrist.com/pcc/pharmacogenetic-guided-treatment-for-neuropsychiatric-disorders

17. Oslin DW, Chapman S, Duvall SL, Gelernter J, Ingram EP, Kranzler HR, et al. Study design and implementation of the PRecision Medicine In MEntal health Care (PRIME Care) Trial. Contemp Clin Trials. 2021 Feb;101:106247.

18. Perlis RH, Mehta R, Edwards AM, Tiwari A, Imbens GW. Pharmacogenetic testing among patients with mood and anxiety disorders is associated with decreased utilization and cost: A propensity-score matched study. Depress Anxiety. 2018 Oct;35(10):946–52.

19. Poster Session III: Wednesday, December 9, 2015. Neuropsychopharmacology. 2015 Dec;40(S1):S443–611.

20. Roe N, Passariello C, Brown L, Li J, Jablonski M, Dechairo BM. 170 Prospective Evaluation of the Economic Utility of Combinatorial Pharmacogenomics in Generalized Anxiety Disorder and Major Depressive Disorder. CNS Spectr. 2018 Feb;23(1):99–99.

21. Prada RM, Beltran AJ, Cardenas RN, Melo VE. 22nd International Abstracts Book. Value Health. 2017 May;20(5):A1–383.

22. Sicras-Mainar A, Guijarro P, Armada B, Blanca-Tamayo M, Navarro-Artieda R. Influence of the CYP2D6 Isoenzyme in Patients Treated with Venlafaxine for Major Depressive Disorder: Clinical and Economic Consequences. De Castro F, editor. PLoS ONE. 2014 Nov 4;9(11):e90453.

23. Tanner JA, Brown LC, Yu K, Li J, Dechairo BM. <p>Canadian Medication Cost Savings Associated with Combinatorial Pharmacogenomic Guidance for Psychiatric Medications</p>. Clin Outcomes Res. 2019 Dec 9;11:779–87.

24. Winner JG, Carhart JM, Altar CA, Goldfarb S, Allen JD, Lavezzari G, et al. Combinatorial pharmacogenomic guidance for psychiatric medications reduces overall pharmacy costs in a 1 year prospective evaluation. Curr Med Res Opin. 2015 Sept 2;31(9):1633–43.

25. Yep T, Devine B. A cost utility analysis of CYP2D6 pharmacogenetic guided dosing versus standard dosing of risperidone for treatment of schizophrenia. Value Health. 2014 May;17(3):A217.

26. Abushanab D, Mohammed S, Abdel-latif R, Al-Muftah W, Ismail SI, Al Hail M, et al. Cost-effectiveness analysis of genotype-guided optimization of major depression treatment in Qatar. J Pharm Policy Pract. 2024 Dec 31;17(1):2410197.

27. Berm EJJ, Gout-Zwart JJ, Luttjeboer J, Wilffert B, Postma MJ. A Model Based Cost-Effectiveness Analysis of Routine Genotyping for CYP2D6 among Older, Depressed Inpatients Starting Nortriptyline Pharmacotherapy. Bishopric NH, editor. PLOS ONE. 2016 Dec 29;11(12):e0169065.

28. Carta A, Del Zompo M, Meloni A, Mola F, Paribello P, Pinna F, et al. Cost–Utility Analysis of Pharmacogenetic Testing Based on CYP2C19 or CYP2D6 in Major Depressive Disorder: Assessing the Drivers of Different Cost-Effectiveness Levels from an Italian Societal Perspective. Clin Drug Investig. 2022 Sept;42(9):733–46.

29. Ghanbarian S, Wong GWK, Bunka M, Edwards L, Cressman S, Conte T, et al. Cost-effectiveness of pharmacogenomic-guided treatment for major depression. Can Med Assoc J. 2023 Nov 14;195(44):E1499–508.

30. Girardin FR, Poncet A, Perrier A, Vernaz N, Pletscher M, F. Samer C, et al. Cost-effectiveness of HLA-DQB1/HLA-B pharmacogenetic-guided treatment and blood monitoring in US patients taking clozapine. Pharmacogenomics J. 2019 Apr;19(2):211–8.

31. Groessl EJ, Tally SR, Hillery N, Maciel A, Garces JA. Cost-Effectiveness of a Pharmacogenetic Test to Guide Treatment for Major Depressive Disorder. J Manag Care Spec Pharm. 2018 Aug;24(8):726–34.

32. Hornberger J, Li Q, Quinn B. Cost-effectiveness of combinatorial pharmacogenomic testing for treatment-resistant major depressive disorder patients. Am J Manag Care. 2015 June 1;21(6):e357-365.

33. Lopez-Saavedra J, Abad-Santos F. Cost-effectiveness of pharmacogenetic screening in the management of major depressive disorder in the Spanish Healthcare System. J Affect Disord. 2024 Nov;365:597–605.

34. Najafzadeh M, Garces JA, Maciel A. Economic Evaluation of Implementing a Novel Pharmacogenomic Test (IDgenetix®) to Guide Treatment of Patients with Depression and/or Anxiety. PharmacoEconomics. 2017 Dec;35(12):1297–310.

35. Ninomiya K, Saito T, Okochi T, Taniguchi S, Shimasaki A, Aoki R, et al. Cost effectiveness of pharmacogenetic-guided clozapine administration based on risk of HLA variants in Japan and the UK. Transl Psychiatry. 2021 July 7;11(1):362.

36. Ninomiya K, Saito T, Ikeda M, Iwata N, Girardin FR. Pharmacogenomic-guided clozapine administration based on HLA-DQB1, HLA-B and SLCO1B3-SLCO1B7 variants: an effectiveness and cost-effectiveness analysis. Front Pharmacol. 2022 Oct 14;13:1016669.

37. Rejon-Parrilla JC, Nuijten M, Redekop WK, Gaultney JG. Economic evaluation of the use of a pharmacogenetic diagnostic test in schizophrenia. Health Policy Technol. 2014 Dec;3(4):314–24.

38. Skokou M, Karamperis K, Koufaki MI, Tsermpini EE, Pandi MT, Siamoglou S, et al. Clinical implementation of preemptive pharmacogenomics in psychiatry. eBioMedicine. 2024 Mar;101:105009.

39. Sluiter RL, Kievit W, van der Wilt GJ, Schene AH, Teichert M, Coenen MJH, et al. Cost-Effectiveness Analysis of Genotype-Guided Treatment Allocation in Patients with Alcohol Use Disorders Using Naltrexone or Acamprosate, Using a Modeling Approach. Eur Addict Res. 2018;24(5):245–54.

40. Sluiter RL, Janzing JGE, Van Der Wilt GJ, Kievit W, Teichert M. An economic model of the cost-utility of pre-emptive genetic testing to support pharmacotherapy in patients with major depression in primary care. Pharmacogenomics J. 2019 Oct;19(5):480–9.

41. Tanner JA, Davies PE, Overall CC, Grima D, Nam J, Dechairo BM. Cost–Effectiveness of Combinatorial Pharmacogenomic Testing for Depression from the Canadian Public Payer Perspective. Pharmacogenomics. 2020 June;21(8):521–31.

42. Ter Hark SE, Kievit W, Hannink G, Vos CF, Spijker J, Van Der Meij A, et al. Genotype-Specific Tricyclic Antidepressant Dosing in Patients With Major Depressive Disorder: A Trial-Based Economic Evaluation. Value Health. 2025 Nov;28(11):1714–21.

43. Chiou CF, Hay JW, Wallace JF, Bloom BS, Neumann PJ, Sullivan SD, et al. Development and Validation of a Grading System for the Quality of Cost-Effectiveness Studies. Med Care. 2003 Jan;41(1):32.

44. Karamperis K, Koromina M, Papantoniou P, Skokou M, Kanellakis F, Mitropoulos K, et al. Economic evaluation in psychiatric pharmacogenomics: a systematic review. Pharmacogenomics J. 2021 Aug;21(4):533–41.

45. Berm EJJ, de Looff M, Wilffert B, Boersma C, Annemans L, Vegter S, et al. Economic Evaluations of Pharmacogenetic and Pharmacogenomic Screening Tests: A Systematic Review. Second Update of the Literature. PLoS ONE. 2016 Jan 11;11(1):e0146262.

46. Djalalov S, Musa Z, Mendelson M, Siminovitch K, Hoch J. A review of economic evaluations of genetic testing services and interventions (2004–2009). Genet Med. 2011 Feb 1;13(2):89–94.

47. Wong WB, Carlson JJ, Thariani R, Veenstra DL. Cost effectiveness of pharmacogenomics: a critical and systematic review. PharmacoEconomics. 2010;28(11):1001–13.

48. Zhu Y, Swanson KM, Rojas RL, Wang Z, St. Sauver JL, Visscher SL, et al. Systematic review of the evidence on the cost-effectiveness of pharmacogenomics-guided treatment for cardiovascular diseases. Genet Med. 2020;22(3):475–86.

49. Bagepally BS, Chaikledkaew U, Chaiyakunapruk N, Attia J, Thakkinstian A. Meta-analysis of economic evaluation studies: data harmonisation and methodological issues. BMC Health Serv Res. 2022 Feb 15;22(1):202.

50. Popay J, Roberts H, Sowden A, Petticrew M, Arai L, Rodgers M, et al. Guidance on the conduct of narrative synthesis in systematic reviews: A product from the ESRC Methods Programme [Internet]. Lancaster University; 2006 [cited 2025 July 2]. Available from: http://rgdoi.net/10.13140/2.1.1018.4643
